# Applying Bayesian Multilevel Modeling to Single Trial Dynamics: A Demonstration in Aversive Conditioning

**DOI:** 10.1002/hbm.70360

**Published:** 2025-09-27

**Authors:** Andrew H. Farkas, Judith Cediel Escobar, Faith E. Gilbert, Christian Panitz, Mingzhou Ding, Andreas Keil

**Affiliations:** ^1^ University of Florida Gainesville Florida USA; ^2^ University of Bremen Bremen Germany

**Keywords:** Bayesian, differential conditioning, generalization, multilevel‐models, single‐trials, steady‐state visually evoked potential

## Abstract

Aversive conditioning changes visuocortical responses to conditioned cues, and the generalization of these changes to perceptually similar cues may provide mechanistic insights into anxiety and fear disorders. Yet, as in many areas of cognitive neuroscience, testing hypotheses about trial‐by‐trial dynamics in conditioning paradigms is challenged by poor single‐trial signal‐to‐noise ratios (SNR), missing trials, and inter‐individual differences. The present technical report demonstrates how a state‐of‐the‐art Bayesian workflow can overcome these issues, using a preliminary sample of simultaneously recorded EEG‐fMRI data. A preliminary group of observers (*N* = 24) viewed circular gratings varying in orientation, with only one orientation paired with an aversive outcome (noxious electric pulse). Gratings were flickered at 15 Hz to evoke steady‐state visual evoked potentials (ssVEPs), recorded with 31 channels of EEG in an MRI scanner. First, the benefits of a Bayesian multilevel structure are demonstrated on the fMRI data by improving a standard fMRI first‐level multiple regression. Next, the Bayesian modeling approach is demonstrated by applying a theory‐driven learning model to the EEG data. The multilevel structure of the Bayesian learning model informs and constrains estimates per participant, providing an interpretable generative model. In the example analysis provided in this report, it showed superior cross‐validation accuracy and provided insights into participant‐level learning dynamics. It also isolated the generalization effects of conditioning, providing improved statistical certainty. Lastly, missing trials were interpolated and weighted appropriately using the full model's structure. This is a critical aspect for single‐trial analyses of simultaneously recorded physiological measures because each added measure will typically increase the number of trials missing a complete set of observations. The present report aims to illustrate the utility of this analytical framework. It shows how models may be iteratively built and compared in a modern Bayesian workflow. Future models may use different conceptualizations of learning, allow integration of clinically relevant factors, and enable the fusion of different simultaneous recordings such as EEG, autonomic, behavioral, and hemodynamic data.

## Graphical abstract

A Bayesian model had superior cross‐validation accuracy of ssVEPs while also being more informative. Left, Model 2 holds the effect of cue constant across participants. Right, the learning model (3) better isolates the aversive conditioning and generalization by allowing learning rates to differ per participant.

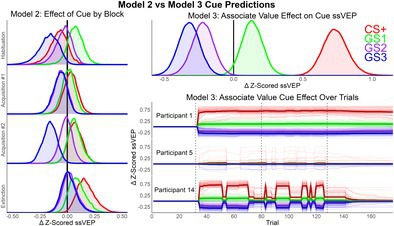

## 1 Introduction

### 1.1 Overview of Technical Report

Cognitive neuroscience is challenged by various sources of noise inherent in noninvasive neuroimaging data. This often prevents researchers from reliably measuring responses from individual participants and trials, crucial for testing and advancing theoretical models of high granularity. The main objective of this technical report is to show how the well‐established approach of Bayesian multilevel modeling can address these issues. By recognizing and specifying the multilevel structure of the data through the priors, information is more efficiently shared across levels of observation, improving model fit and enabling the development of more useful and complex theoretical models. This paper demonstrates this in two examples using a preliminary sample of simultaneous EEG‐fMRI data recorded during aversive generalization conditioning. First, a typical fMRI first‐level multiple regression is improved by adding a multilevel structure. Second, the well‐established Rescorla‐Wagner model of classical conditioning is extended with a multilevel structure to quantify dynamic changes in EEG responses. While these insights are specific to the field of aversive conditioning, the approach is readily generalized to many fields of cognitive neuroscience as it enables researchers to systematically and quantitatively compare, test, and advance theoretical models.

### 1.2 Neuroimaging of Aversive Conditioning

Experience is known to change the tuning of visuocortical neurons, heightening the sensitivity to simple features that selectively predict motivationally relevant outcomes (Li and Keil 2023; Shuler and Bear 2006). This process is readily studied by means of Pavlovian generalization conditioning (Lissek et al. 2014, 2012). In this paradigm, stimuli varying in similarity are presented, but only one stimulus (the CS+) is consistently paired with the unconditioned stimulus (US, e.g., loud noise, electric shock), whereas other stimuli (generalization stimuli, GSs) are never paired. A test phase tends to follow an initial conditioning phase (Lonsdorf et al. 2017). Typical findings include that response variables such as skin conductance or pupil diameter are heightened when viewing the CS+, and that this enhancement is partly transferred to the GSs, as a function of their similarity with the CS+ (Ahrens et al. 2016). Neuroimaging studies of aversive generalization conditioning have found similar activation patterns in brain structures such as the amygdala or anterior insula (Dunsmoor and Murphy 2015; Lissek et al. 2014). Studies interested in inter‐individual differences have further observed that relatively heightened responses to GSs, referred to as overgeneralization, are often associated with elevated anxiety and negative affect (e.g., Lissek et al. 2008). Together, these observations highlight the foundational role of generalization learning for adaptive behavior.

What is currently not understood is how generalization learning emerges over time and how it shapes different physiological systems as observers learn to predict the aversive outcome. To do this, the present study simultaneously recorded fMRI and EEG steady‐state visually evoked potentials (ssVEPs). Such ssVEPs (Norcia et al. 2015; Wieser, Miskovic, and Keil 2016, Wieser, Reicherts, et al. 2016) are one of the most robust methods for indexing how aversive learning emerges in the visual brain on a trial‐by‐trial basis. The ssVEP is evoked when a visual stimulus is flickered at a specific rate (here: 15 Hz), eliciting a strong electrocortical signal in sensors over the visual cortex that oscillates at the same rate. Readily recorded from sensors placed over the scalp, the amplitude of electrical activity at the target frequency can be quantified in the frequency domain. The ssVEP originates primarily in calcarine and peri‐calcarine (lower‐tier) visual areas, making it most pronounced at occipital recording sites. Over the past decades, we and others have established that the amplitude of the ssVEP varies with selective attention to motivationally relevant stimuli and that this modulation originates in visual and fronto‐parietal cortical regions (Wieser and Keil 2011, 2020). The ssVEP signal represents periodic repetitions at a known specific frequency, and thus can be collapsed into one bin of a frequency‐domain representation such as a Fourier spectrum, yielding a high signal‐to‐noise ratio (Müller and Hillyard 2000; Petro and Keil 2015; Regan 1972). This property makes the ssVEP uniquely suitable for estimating robust single‐trial amplitude values (Figueira et al. 2022). Such an ability is in contrast with most measures used in human neuroscience, which are dependent upon trial averaging to eliminate noise (Fabiani et al. 2007). A high signal‐to‐noise ratio is crucial for studies of learning in which dynamic trial‐by‐trial changes are expected. Thus, in the present study, it was paired with simultaneous fMRI recording to measure the relevant mechanisms.

Yet, problems remain that can be addressed by the Bayesian approach: Like in many neurophysiological studies, inference on aversive conditioning dynamics is challenged by missing trials and inter‐individual differences. It is common for various artifacts to cause some trials to be unusable, leading to “ragged” data sets with missing observations. This situation further deteriorates as more physiological measures are recorded simultaneously because even fewer trials will have a complete set of observations. Possibly more concerning is the difficulty in modeling inter‐individual differences. It is expected that participants will learn aversive pairings at different rates, and aspects like generalization may depend on observer traits such as anxiety. Although this can be the explicit purpose and focus of a conditioning study, individual differences become another source of noise if not modeled correctly (Hedge et al. 2018). Bayesian multilevel models address these problems in which granular estimates (e.g., individualized learning rates) are both informed and constrained by a hierarchical structure of priors (e.g., the average learning rate) as previously described. Another specific benefit for trial‐dependent physiology studies is that missing trials can be specified as additional parameters to be estimated. This results in missing trials being interpolated not as a single point but as a distribution informed by the full model's structure and thus reflecting the appropriate amount of statistical uncertainty.

In the present example fMRI analyses, we demonstrate that standard fMRI first‐level multiple regression is improved by the use of multilevel priors. First, it is shown that statistical certainty and motion correction are improved for region‐of‐interest (ROI) level estimates. Secondly, it is shown that the multilevel pooling improves the separation of overlapping hemodynamic responses related to US shocks versus the CS+ cue.

Next we demonstrate the fit of three models on the ssVEP data. We chose these data for the present demonstration because they illustrate some of the problems inherent in multimodal recordings that are readily addressable by Bayesian multilevel analyses: missing data, pronounced inter‐individual variability, and the need for single‐trial level information. Each subsequent model has increased complexity, starting as a description of overall trends in the trial‐wise ssVEP amplitudes to the final model (Model 3) that estimates individual participant learning rates using a version of the Rescorla‐Wagner learning rule. In this rule, memory updating (learning) is quantified as a person's prediction error on a given experimental trial. The learning rate parameter (*α*) quantifies how new experience is weighted as predictions are updated. This model posits that as the prediction error increases, changes in associative strength are larger and as the prediction error decreases, less learning takes place (Rescorla and Wagner 1972; see Equation (1)).

All three EEG models compared in the present report interpolate missing trials and model inter‐individual differences in the adaptation (reduction) of the ssVEP amplitude over the course of the study. After outlining EEG preprocessing and model equations, the models are compared in terms of cross‐validation accuracy. Posterior distributions are used to visualize and interpret the effects of interest, which include skewed and bounded learning rates that would be difficult to fit and interpret with Frequentist multilevel models. Lastly, the Bayesian interpolation of missing trials is visualized. This latter feature is especially relevant for multimodal or multi‐variable imaging studies, in which multiple measures (e.g., EEG, fMRI‐BOLD, Heart Rate, Pupil diameter, etc.) are recorded concurrently.

## 2 Methods

### 2.1 Participants

Participants were recruited from the University of Florida Psychology research pool and compensated with course credit. Because this was a simultaneous EEG‐fMRI paradigm, data collection was more complicated, with more points of failure compared to studies that only record EEG or fMRI. A total of 38 participants were run through the complete conditioning paradigm. Four were removed from both imaging modalities because of poor data quality from excess movement. Another participant was removed due to vision acuity issues limiting their ability to see the cues or read rating instructions. The last removal was a participant who fell asleep during the study. Of the remaining participants, 8 had unusable EEG because of equipment malfunctions. Thus, the preliminary EEG sample comprised 24 participants, with 18 being female and 8 identifying as Hispanic. The average age of the sample was 19.9 (SD = 1.78). The self‐identified race of the sample was 18 White, 3 Asian, 2 Multiracial, with one participant not reporting race. For fMRI recordings, the same 24 participants could not be used because 6 of these individuals had movement exceeding our predefined thresholds. So, the first 24 participants with less than 25% of the fMRI volumes censored through the AFNI standard preprocessing, or less than 2 mm or 2 degrees of total motion, were selected as a demonstration sample. This fMRI sample comprised 15 females, 8 identifying as Hispanic, and the average age was 20.5 (SD = 2.53). The racial breakdown was 18 identifying as White, 4 as Asian, and 2 as Multiracial. All participants gave written informed consent using forms approved by the University of Florida Institutional Review Board, which approved all procedures.

### 2.2 Procedure

After arriving at the data collection building, participants were guided to a quiet preparatory room. After consenting, participants were then screened for the MRI scanning procedure to ensure no safety or medical concerns were present. After all the equipment was fitted, but prior to entering the scanner, participants completed a series of questionnaires and demographic information.

Researchers then added Biopac spectra360 GEL104 to two EL509 MR‐safe dry adhesive electrodes and applied the gelled electrodes to the participant's left outer ankle. Electrodes were positioned two finger widths above the left ankle bone and slightly anterior to the fibula. The electrical stimulus (0.067 s in duration) was administered using the Biopac STMISOC system connected to the STM100C stimulator module, with LEAD108 connecting to the EL509 electrodes. A shock workup procedure was then implemented, where the electrical stimulus intensity was increased until participants stated that it was “unpleasant but not painful, and still tolerable”. This procedure was repeated twice. After obtaining the stimulus threshold, participants were then asked to rate a series of 5 shocks in a row on a scale of 1 to 9, with 9 being extremely unpleasant and 1 being not unpleasant at all. After the final shock, the participant was asked if the stimulus was still unpleasant but tolerable. The average shock level was 7.26 mA (SD = 5.0, min = 1 mA, max = 50 mA).

After the shock tuning, participants were fit with a 32‐channel MR‐compatible EEG system (BrainAmp MR; Brain Products). Gel was applied using blunt tip syringes and cotton swabs until impedance values were below 10 kΩ or as low as possible in accordance with the Brain Product recommendations. The electrode for measuring ECG was placed on the participant's back slightly to the left of the spine, while tucking the chin to allow enough slack for the cable while lying down in the scanner. A Siemens Head/Neck 64 coil was placed over the participant's head, and the cable connecting the EEG to the amplifier was fed through the back of the head coil. A mirror attached to the head coil allowed participants to view the presentation monitor, which was located 70 in. away from the participant.

Participants were then led to the 3 T chamber and given earplugs to reduce any discomfort from the noise of the scanner. In their right hand, participants were given a clicker with a left and right button for making ratings throughout the study. After each block, participants were shown a Gabor pattern and were given 6 s to make their rating. They could click left and right to indicate their rating from the middle of an 11‐point Likert scale for ratings of hedonic valence and arousal. Additionally, participants were also asked to rate their expectancy of a shock for each pattern from 0 to 100% in 10% increments. In the present manuscript, the ratings are only briefly mentioned because other models better demonstrate the strengths of the multilevel method. As is discussed later, while ratings had a modest group‐level effect, they did not fit well at the per‐participant level. The ratings and auxiliary models that used ratings to fit the ssVEP are more thoroughly explained in the Supporting Information.

A two‐way communication microphone was utilized to maintain communication between the participant and researcher for instructions and in case of emergency. Following the task, participants were removed from the chamber and asked to rate the unpleasantness of the shock the last time they felt it. Participants were also asked if they thought there was any relationship between the patterns and the electric stimulus. Only participant 10 was unable to state the correct conditioning relationship. After debriefing, participants were thanked and compensated for their time.

### 2.3 Conditioning Paradigm

This task involved a fear conditioning paradigm with a sequential design involving 4 blocks of Habituation, Acquisition #1, Acquisition #2, and Extinction. In this design, flickering Gabor patches with different grating orientations were presented for 2 s with a random ITI drawn from a truncated exponential distribution with a minimum of 5.5 s, a mean of 7, and a maximum of 15.4. There were four unique Gabor cues used in the task that were identical except in the orientation they were presented. The CS+ was pseudo‐randomly counterbalanced between participants to be either 15° or 75° counterclockwise from vertical. The generalization stimuli (GS) are labeled in relation to the orientation of the CS+, such that if the CS+ is 15°, then GS1 is 35°, GS2 is 55°, and GS3 is 75°. Each cue took up 5° of visual angle and had a spatial frequency of 3.5 cycles per degree of visual angle.

For each block, the same Gabor cue presentations were randomized, and the same cue could only be presented twice consecutively. After each block, participants used the clicker to rate valence, arousal, and shock expectancy per cue as already described in the procedure. The first block was Habituation, in which no US shocks were given, and each Gabor orientation was presented 8 times for a total of 32 trials. Each subsequent block contained 12 cue presentations for a total of 48 trials. For the first Acquisition block, the first 6 CS+ presentations had a reinforcement rate of 100%, which means they were always paired with US ankle shock. For paired CS+ trials, the shock was delivered during the final 0.067 s of the cue and then co‐terminated with the stimulus. The first 6 CS+ trials were also boosted such that no more than 2 GS‐separated CS+ trials. Following the initial CS+ trials, the reinforcement rate was lowered to 50% for the remainder of Acquisition Block 1 as well as for Acquisition Block 2. The last block was Extinction in which the CS+ cue was no longer paired with the US.

### 2.4 Data Acquisition and Data Reduction

The MRI data was collected using a 3 T/60 cm Siemens Prisma scanner. A T1 structural image was obtained by means of the mprage protocol; TR = 2.3 S, TE = 2.32 Ms., Flip angle = 8°, FOV = 240 mm, voxel size = 0.9 mm^3^. The fMRI was acquired via an echo planar imaging sequence tuned to interfere less with the EEG recording; TR = 2 S, TE = 22.4 Ms., Flip angle = 40°, FOV = 212 mm, voxel size = 1.8 mm^3^, slices = 64, multi‐band acceleration = 2. Additionally, two distortion mapping scans were acquired with the phase encoding direction (anterior to posterior) reversed in one of the scans to estimate and correct for field inhomogeneities. The fMRI was preprocessed via an AFNI (Cox 2012) pipeline designed with the recommended afni_proc.py (Reynolds et al. 2024) command which can be found in the Supporting Information. This included slice time alignment, inhomogeneity correction, volume alignment and registration to the T1 structural, warping to the MNI152C asymmetric template, 4 mm full‐width at half‐maximum blur, and a scaling of each voxel to percent difference. The quality of each participant was checked via built‐in AFNI tools (Reynolds et al. 2023). Participants were excluded if over 25% of their data was censored or total movement exceeded 2 mm or 2 degrees. Censored fMRI volumes were found via the suggested temporal outliers threshold of 5% and 0.3 mm approximated change. The BOLD time series was then extracted for the left ventral anterior insula from the human connectome volumetric atlas (Glasser et al. 2016). This region has been implicated in previous conditioning research (e.g., Sperl et al. 2019). Finally, each voxel's BOLD time series per participant was detrended by a 5th order 0.0065 Hz high‐pass filter, applied in the forward and reverse directions.

The EEG was acquired using a 32‐channel MR‐compatible EEG system (BrainAmp MR; Brain Products). The system uses 31 Ag/AgCl scalp electrodes positioned at the standard 10–20 positions, with the reference at the FCz location and an online bandpass filter from 0.1 to 250 Hz. The 32nd channel records ECG from a channel positioned on the back, slightly to the left of the spine. Processing was accomplished with custom MATLAB scripts using the EEGLAB toolbox and plugins as well as functions from the FreqTag toolbox (Figueira et al. 2022). The data and code used for processing, models, analyses, and figures can be found on this project's OSF page (https://osf.io/hfbjp/).

Two main artifacts affect EEG data in an fMRI scanner: gradient artifacts from the functional scanning and cardioballistic artifacts accentuated by the static magnetic field (Debener et al. 2006). To correct for these artifacts, a mix of specialized equipment and established processing procedures was implemented. More specifically, it is necessary to ensure that there are accurate markers for gradient switching and heart beats, such that these repeated sources of error can be subtracted out (Allen et al. 2000; Niazy et al. 2005). The brain products EEG system uses specialized hardware to receive TTL pulses for each fMRI repetition time (TR), which corresponds to each collected volume. These TRs are passed to a synchronization box to align the MRI and EEG systems' clocks and are then written into the EEG file as markers in the acquired data. The distance between TR pulses was confirmed to be accurate to the 4th decimal place. The data were acquired at 5 kHz, as higher sample rates help in correcting for artifacts. The gradient artifact correction was applied using the fmrib_fastr function described in Niazy et al. (2005), implemented from an EEGlab toolbox script. The function is designed to align volumes, subtract a moving average, and then to subtract principal components of residual artifacts. The PCA removal was implemented but led to no differences in the overall pattern of effects for all three models and thus was omitted from the final pipeline used for the results reported below. Because no PCAs were removed, gradient artifacts were handled by the standard subtraction method as described in Allen et al. (2000). The data were then down‐sampled to 500 Hz, and the Niazy et al. (2005) algorithm was used to find the R peaks from in the ECG signal, followed by applying the fmrib_pas function, which identifies PCA components in the EEG that correspond to cardio‐ballistic artifacts. Three principal components were removed to correct for the cardio‐ballistic artifact. Processing the data without any cardio‐ballistic artifact correction led to near‐identical results for all three models, with an average of eight more trials retained per participant. The correction was applied in the present analysis pipeline because it is standard practice (Petro et al. 2017). The data were then filtered with a Hamming windowed sinc FIR with a high‐pass of 0.05 Hz and low pass of 40 Hz (3‐dB points), implemented with the pop_eegfiltnew function from EEGlab.

The data were then inspected by two members of the research team independently by scrolling through the recordings and considering the ssVEP spectrum for each trial. Bad channels were removed based on agreement between the research team and are listed per participant in the preprocessing script on OSF (a002EEG_prepro.m; https://osf.io/hfbjp/). No participants had Oz as a bad channel, which was the only sensor analyzed. The preprocessing script then performed an ICA using the SOBI algorithm from the pop_runica function in EEGlab (Tran et al. 2009). Removing the ICA components related to eye‐blinks led to no differences across the models, so ultimately no ICA components were removed from the final data set. Missing channels were interpolated via a spherical spline using the pop_interp function. The data were then re‐referenced from the FCz location to an average reference. The pop_clean_rawdata function was then used to identify artifact‐contaminated sections of the data. The channel rejection options were disabled. Additionally, no high‐pass filtering was applied. Artifactual bursts were identified using a threshold of 20 standard deviations, and those sections were trimmed from the data set. The Euclidean distance was used to identify windows in which 25% of the window was affected. In a final step, the data were transformed to a current source density (CSD) representation, implemented via the current_source_density function from the ERPLAB toolbox (Lopez‐Calderon and Luck 2014).

Retained single trials were extracted from each participant via the pop_epoch function. Trials were segmented from cue onset to offset (2000 ms duration). The oscillatory power for the 15 Hz ssVEP driving frequency was quantified via by means of a Discrete Fourier Transform (DFT) implemented from the freqtag_FFT3d function from the FreqTag toolbox (Figueira et al. 2022), applied to the entire 2000 ms segment for each trial, with no windowing applied, leading to a spectral frequency resolution of 0.5 Hz. This was done for the preprocessed data and was compared against an alternative version of the pipeline in which a sliding window analysis was used to further isolate the ssVEP frequencies (Morgan et al. 1996; Wieser, Miskovic, and Keil 2016, Wieser, Reicherts, et al. 2016; Figueira et al. 2022). Ultimately, the DFT was used for simplicity because the sliding window analysis led to near equivalent results across the three models. Finally, the mean amplitude for the 15 Hz frequency was extracted for each retained trial from the Oz sensor, Z‐scored within‐participant, and was used as the dependent measure for all Bayesian multilevel models (referred to as *Amplitude* in the equations).

### 2.5 Bayesian Models and Analyses

Two aspects of the Bayesian workflow are demonstrated below. The first is to detail how a Bayesian multilevel structure is generally beneficial by showing how it improves a standard fMRI multiple regression. The second aim is to further demonstrate the modeling approach by going beyond a descriptive model to fitting a theoretical model to the EEG‐ssVEP data. The current analyses rely on the theoretical approach sometimes described as Bayesian workflow (Gelman et al. 2014, 2020; McElreath 2020; Gabry et al. 2019; Schad et al. 2021), which involves building competing generative models that act as specific hypotheses. This process aligns with the notion of systemic/programmatic research, in which theories are iteratively created, made more specific, or discarded over time (Popper 2005; Lakatos 1970; Kuhn 1997). Another objective of this approach is to detail uncertainty, which has been argued is more informative than point‐estimate significance testing (McElreath 2020; Wasserstein et al. 2019). For these reasons, comparing models and visualizing uncertainty are prioritized in the present report.

The necessary elements for a Bayesian model of empirical data include specifying (1) a model for how the data is distributed (a sampling model that captures likelihoods) and (2) priors for all unknown parameters. In line with best practices (Gelman et al. 2014; McElreath 2020), the present models were built iteratively in open‐source code. Also in line with recommendations, priors were selected to be either uninformative but on a realistic scale for the data, or as a regularizing multilevel prior (Gelman et al. 2012). Visualizations and rationale for the uninformative priors can be found in the Supporting Information on OSF webpage. The models were specified and estimated in the Stan statistical programming language (Stan Development Team 2024) using a variant of the Hamiltonian Monte Carlo sampling method (Duane et al. 1987; Hoffman and Gelman 2014). For the EEG models, a total of 80,000 samples were taken to estimate the posteriors across 8 Markov Chains on a laptop with an AMD Ryzen AI 9 HX 370 CPU. The fMRI models were more computationally expensive as they required a 1070 × 1070 Cholesky matrix decomposition per leap frog step per participant. Thus, fMRI models were fit using 5000 samples across 5 chains on the University of Florida HiPerGator cluster, in which 24 CPU cores were used per chain. For all models, the chains converged, with the *R‐hat* metric well below 1.05 for all estimated parameters.

The two fMRI models presented use the same statistical model as AFNI's 3dREMLfit. This analysis utilizes the standard first‐level multiple regression used in most fMRI analyses in which a design matrix of predicted hemodynamic responses per stimulus and metrics of brain movement are fit to the BOLD timeseries in a regression. However, to provide a less biased error estimate, 3dREMLfit estimates the proportion of temporal correlation between volumes via a Toeplitz covariance matrix (https://afni.nimh.nih.gov/pub/dist/doc/misc/3dREMLfit/3dREMLfit_mathnotes.pdf; last access July 31, 2025). This model specification was replicated in Stan code for both fMRI Models 1 and 2. Model 1 fits each free parameter per participant as would be done in the first‐level of most fMRI analyses. Model 2 differs in that every parameter per participant receives a multilevel prior in which the average is simultaneously estimated. The design matrices were taken from the AFNI preprocessing pipeline. The predicted hemodynamic responses in the design matrices were found via the AFNI Block (2, 1) function which convolves a typical hemodynamic response by a square of 2 s in duration (first function argument) with a fixed maximum height of 1 (second argument). This is a typical analysis in which the solved coefficient estimates the percent change in the hemodyamic response to the stimulus. An additional column was added for the shock stimulus using the Block (0.1, 1) reflecting the shorter duration of the US. In total, there were 19 predictor parameters estimated per participant: 12 for each cue per phase of the study (habituation, acquisition, and extinction), 1 for the US shock, and the last 6 were the estimated movement (*x*, *y*, and *z* axes) and rotation (roll, pitch, and yaw).

While the fMRI analysis is a primarily descriptive analysis, the ssVEP analyses demonstrate how theoretical models can also be fit and compared. Eight ssVEP models were fit to the data, of which only three are presented in the manuscript for simplicity and interpretability. Three of the unreported models used slightly different parametrizations of learning ratings, leading to lower cross‐validation accuracy, but no notable changes in model interpretations. The other two models not reported in the main text used individual participant ratings of arousal and US expectancy per cue and block to predict ssVEP amplitude. There was a marginal relationship between arousal ratings, but poorer cross‐validation accuracy compared to the three presented models. The code and discussion of these five models can be found on the OSF project accompanying this manuscript at https://osf.io/hfbjp/.

There are several elements that were held constant for interpretability between the models. The first is that all models were fit to observed and the estimated missing trials. So an array was formed with missing and observed ssVEP amplitudes, allowing for Bayesian imputation in which missing trials are simulated based on the model's predictions.

(1) $$\text{Amplitude}\left[1:\mathrm{all}\text{possible trials}\right]$$

(2) $$\text{Amplitude}\left[\text{indicesObserved}\right]=\text{AmplitudeObserved}$$

(3) $$\text{Amplitude}\left[\text{indicesMis}\sin g\right]=\text{AmplitudeMis}\sin g$$

Continuing, each model uses the typical normal distribution for the likelihood function, such that error around each trial's predicted amplitude (*μ*) is expected to be normally distributed with a standard deviation of *σ*. The *σ* parameter was allowed to differ between participants. Equation (4) is how this is written in statistical notation and the Stan models, where “*i*” loops through each trial and “participant” is an array of integers specifying the current participant:

(4) $$\text{Amplitude}\left[i\right]\sim \text{Normal}\left(\mu \left[i\right],\sigma \left[\text{participant}\left[i\right]\right]\right)$$

The parameter for the error per participant (*σ*) was given a multilevel prior to regularize it by the average (*σAverage*). Error posteriors closer to zero suggest better predictions per trial. Because of the limited sample size, the regularizing multilevel prior was specified as a Student‐T distribution as opposed to a normal distribution. The prior for *σAverage* was centered at the initial *Z*‐scored error of 1.

(5) $$\sigma \left[1:\text{nParticipants}\right]\sim \text{StudentT}\left(\mathrm{df}=\left(\text{nParticipants}-1\right),\text{σAverage},\tau \right)$$

(6) $$\text{σAverage}\sim \text{Normal}\left(1,0.5\right)$$

Deviation parameters such as *τ* could be very close to zero if the error per trial between participants (*σ*) is similar between participants. This can make it difficult to sample *τ* with the Stan Hamiltonian Monte Carlo method. For this reason, many of the deviation parameters were specified as an unbounded parameter that were then exponentiated to get back to the correct positive‐only scale required. So as shown, only the *Raw* variant received a prior and was sampled directly before being transformed. This allows for posteriors to be sampled more effectively because they are flatter even if they become very close to zero as the model is fit. As mentioned, visualization and rationale for these priors can be found in the Supporting Information.

(7) $$\tau =\exp\left(\text{τRaw}\right)$$

(8) $$\text{τRaw}\sim \text{Normal}\left(-3,1\right)$$

#### 2.5.1 Model 1: Only Adaptation Over Trials With No Effect of Cue

Prior conditioning studies and other ssVEP studies have observed that the amplitude of visuocortical responses may decrease over the course of the experimental session, thought to reflect several neurophysiological and behavioral factors, including visuocortical adaptation, increasing spontaneous alpha oscillations, and participant fatigue (Norcia et al. 2015; Liu et al. 2012). Thus, we first modeled an adaptation gradient of the ssVEP amplitude over the course of the study that could differ between participants. This was modeled as a linear effect across trials for each prediction *μ* for all models.

(9) $$\mu \left[i\right]=\mathrm{int}\text{ercept}\left[\text{participant}\left[i\right]\right]+\text{adaptation}\left[\text{participant}\left[i\right]\right]\cdot \text{trialCentered}\left[i\right]+\ldots$$

The adaptation intercept and slope were the only predictors used for Model 1; thus, this model acts as a Null model for comparative purposes. The intercept was centered to the median trial by subtracting 88.5 ((176 + 1)/2) from the trial index to get *trialCentered*. For the same reasons as *τ*, *σIntercept* and *σAdaptation* were estimated via exponentiation of sampled unbounded parameters. Correlations between participants and slopes were found via *ρ*, which is derived from the prior *ρRaw* that is roughly uniform after the hyperbolic tangent transformation. The *ρ* parameter was more necessary in previous versions of the models that did not center the intercept. Both specifications of the intercept led to equivalent results in all models.

(10) $$\begin{array}{c} \left[\begin{array}{c} \mathrm{int}\text{ercept}\left[1:\text{nParticipants}\right]\\ \text{adaptation}\left[1:\text{nParticipants}\right] \end{array}\right]\sim \\ \text{MVStudentT}\left(\mathrm{df}=\left(\text{nParticipants}-1\right),\left[\begin{array}{c} \mathrm{int}\text{erceptAverage}\\ \text{adaptationAverage} \end{array}\right],\Sigma \right) \end{array}$$

(11) $$\Sigma =\left[\begin{array}{cc} \sigma {\text{Intercept}}^{2} & \rho \cdot \text{σIntercept}\cdot \text{σAdaptation}\\ \rho \cdot \text{σIntercept}\cdot \text{σAdaptation} & \sigma {\text{Adaptation}}^{2} \end{array}\right]$$

(12) $$\mathrm{int}\text{erceptAverage}\sim \text{Normal}\left(0,0.2\right)$$

(13) $$\text{adaptationAverage}\sim \text{Normal}\left(0,0.01\right)$$

(14) $$\text{σIntercept}=\exp\left(\text{σInterceptRaw}\right)$$

(15) $$\text{σAdaptation}=\exp\left(\text{σAdaptationRaw}\right)$$

(16) $$\rho =\tanh\left(\text{ρRaw}\right)$$

(17) $$\text{σInterceptRaw}\sim \text{Normal}\left(-1,1\right)$$

(18) $$\text{σAdaptationRaw}\sim \text{Normal}\left(-4.5,0.75\right)$$

(19) $$\text{ρRaw}\sim \text{Normal}\left(0,1\right)$$

#### 2.5.2 Model 2: Mean of Cue per Block

The second model predicts each trial's ssVEP amplitude as a combination of the adaptation slope with an effect of each cue (CS+, GS1, GS2, GS3) per block (4 total: 1 habituation, 2 Acquisition, 1 Extinction). The effect of each cue per block was specified as a 4 × 4 array (*βCue*) and was regularized via a Student‐T distribution and the deviation parameter *σCue*. The mean for all cues was set to zero to allow it to vary from the adaptation regression line per participant. The generalization stimuli differ experimentally in their orientational similarity from the CS+ Gabor patch, such that GS1 is 15 degrees different, GS2 is 30°, and GS3 is 45°. However, this similarity is not explicitly enforced on the *βCue* parameters. All 16 parameters are treated as independent effects from the Student‐T distribution.

(20) $$\begin{array}{cc} \mu \left(i\right)= & \;\mathrm{int}\text{ercept}\left(\text{participant}\left[i\right]\right)\\ & \;+\text{adaptation}\left(\text{participant}\left[i\right]\right)\cdot \text{trial}\left(i\right)\\ & \;+\text{βCue}\left(\mathrm{cue}\left[i\right],\text{block}\left[i\right]\right) \end{array}$$

(21) $$\text{βCue}\sim \text{StudentT}\left(\mathrm{df}=\left(\text{nCues}\cdot \text{nBlocks}\right)-1,0,\text{σCue}\right)$$

(22) $$\text{σCue}=\exp\left(\text{σCueRaw}\right)$$

(23) $$\text{σCueRaw}\sim \text{Normal}\left(-0.5,1.5\right)$$

#### 2.5.3 Model 3: Multilevel Rescorla‐Wagner Inspired Associate Strength

A goal of the present study was to apply models of associative learning to neurophysiological data. The Rescorla‐Wagner model is one of the most well‐established in which the change in associative strength (*ΔCS + Strength*) is modeled as the difference in current associative strength (*CS + Strength*) from a binary variable specifying if the US was paired or not (1 = US or 0 = no US) scaled by a *LearningRate* parameter (Rescorla and Wagner 1972). Both *LearningRate* and *CS + Strength* are constrained to be between 0 and 1.

(24) $$\Delta \text{CSPStrength}\left(i\right)=\text{LearningRate}\cdot \left(\text{paired}\left[i\right]-\text{CSPStrength}\left[i\right]\right)$$

(25) $$\text{CSPStrength}\left(i+1\right)=\text{CSPStrength}\left(i\right)+\Delta \text{CSPStrength}\left(i\right)$$

The Rescorla‐Wagner model has been applied widely (Soto et al. 2023; Esber et al. 2025). What is novel about the present study is the use of multilevel priors to allow it to fit more flexibly to each participant. Model 3 allows two learning rates (for paired and unpaired CS+ trials) per participant, each of which is regularized by a multilevel prior. The accumulated CS+ strength is then used to predict the change in ssVEP amplitude via a weight per cue, which is also regularized by a multilevel prior. The predicted ssVEP per trial is found via the following equation.

(26) $$\begin{array}{cc} \mu \left(i\right)= & \;\mathrm{int}\text{ercept}\left(\text{participant}\left[i\right]\right)\\ & \;+\text{adaptation}\left(\text{participant}\left[i\right]\right)\cdot \text{trial}\left(i\right)\\ & \;+\text{βScaling}\left(\mathrm{cue}\left[i\right]\right)\cdot \text{CSPStrength}\left(i\right) \end{array}$$

A more thorough description of model equations and parameterization can be found in the Supporting Information.

#### 2.5.4 Model Comparison via PSIS‐LOO Cross‐Validation

Models with many parameters can overfit the observed data, leading to interpretations that would not generalize to new data. Cross‐validation is an established method for addressing this issue, in which accuracy is measured on data held out of the model fitting. For Bayesian models, the leave‐one‐out (LOO) cross‐validation accuracy can be estimated with Pareto‐smoothed Importance Sampling (PSIS‐LOO; Vehtari et al. 2024). This measure has been shown to provide equivalent information to ordinary LOO and k‐fold cross‐validation or information criteria statistics (Vehtari et al. 2017). Additionally, the models do not need to be iteratively refit, and diagnostic Pareto‐*k* weights indicate if the cross‐validation measure is valid or is being skewed via outliers. Cross‐validation accuracy was only measured on observed data and not the *AmplitudeMissing* parameters used in model estimation. Out of the 3560 observed ssVEPs, 7 had Pareto‐*k* weights above 0.7, indicating outliers for Model 3. However, the number of estimated effective samples was still high (2121), and excluding these observations has no effect on overall interpretations. For fMRI models, there were no problematic Pareto‐*k* weights.

The PSIS‐LOO method converts the log‐likelihood per observation to an expected log‐likelihood of if the data were held out of the model fitting. Models are compared via the sum of expected log likelihoods known as the expected log‐likelihood predictive density (ELPD). Because log‐likelihoods are on a scale from negative‐infinity (maximum unlikely) to zero (perfect prediction with no uncertainty), a model with a more positive ELPD indicates better cross‐validation accuracy over all observations. Models were compared based on their overall differences in ELPD via built‐in Stan functions. The standard error of each sum or difference of sums was found with the standard equation:

(27) $$\sqrt{N\cdot \mathrm{var}\text{iance}\left(\text{PSIS}-\mathrm{LOO}\right)}$$

The ELPD values were used to further compare models via stacking weights that indicate what linear combination of the models would lead to the best cross‐validation accuracy. During this procedure, the number of effective parameters is estimated, which can give a sense of how multilevel priors regularize the number of degrees of freedom (Vehtari et al. 2017). ELPD was separated per cue, block, and participant to assess at which points models had better cross‐validation accuracy. Finally, the cross‐validation accuracy was further interpreted per participant via LOO‐adjusted *R* ^2^ (variance explained) posteriors. This was accomplished via a Dirichlet weight resampling Bayesian Bootstrap procedure adapted from the loo_R2 function from the rstanarm R package (last accessed 12/30/24, https://raw.githubusercontent.com/stan‐dev/rstanarm/refs/heads/master/R/bayes_R2.R). After resampling, the *R* ^2^ is defined as:

(28) $$\mathrm{LOO}‐R^{2}=1-\frac{\text{variance}\left(\mathrm{LOO}\text{residuals}\right)}{\text{variance}\left(\text{data}\right)}$$

Thus, *R* ^2^ posterior samples can be negative if the model predictions are poor and produce more variance than in the original data.

#### 2.5.5 Posterior Analyses

Not all estimated parameter posteriors are presented in the main text, but they can be found in the Supporting Information. In line with contemporary statistical recommendations (McElreath 2020; Wasserstein et al. 2019), representations of statistical uncertainty are prioritized with posterior visualizations or numerical descriptions as opposed to significance testing. The inner 95% posterior densities, and if they overlap with zero, are reported where applicable to detail the uncertainty of the parameters or posterior contrasts.

For the fMRI models, the predicted mean posterior for the anterior insula was found per participant and for illustration was plotted over the original ROI timeseries for 3 randomly selected participants. This posterior time series was found through matrix multiplication of the design matrix with the estimated 19 predictor parameters per participant. The inner 95% of the posterior per volume was plotted to show differences in statistical certainty between the models. To show how the multilevel structure regularized individual participant estimates, the effect of each cue per phase and US was plotted per participant for both the multilevel and non‐multilevel fMRI models.

The learning rate posteriors per participant were plotted as densities between the their bounded values of 0 and 1. To show how learning rates relate to *CS + strength*, the median and 100 posterior draws of this posterior are also depicted. To understand if specific learning patterns were related to poor model fit, the LOO‐adjusted *R* ^2^ posteriors per model were also depicted by participant in the same figure (Figure 4). To understand the effects of cue and US pairing, the posterior densities for *βCue* from Model 2 and *βScaling* from Model 3 are depicted. In this same figure, the combined effect of *βScaling* with *CS + Strength* is demonstrated via the product of these parameters for three participants (1, 5, and 14) via 100 posterior draws and the full posterior average per cue. To test our hypothesis of aversive generalization in the ssVEP, we performed a linear contrast in the expected direction by subtracting the posterior samples of the generalized stimuli (GS) from the CS+ in order of their similarity to the CS+. This was done per block for the *βCue* parameters for Model 2 and just once for the four *βScaling* weights from Model 3. Lastly, the missing trial interpolation for Model 3 is illustrated by 100 draws for the missing trials in a subsection of the data from participants 1 and 14. These interpolated trials are contextualized with lines representing the average posterior for the mean per cue and the surrounding observed data points.

## 3 Results

### 3.1 Functional MRI Results

**FIGURE 1:**
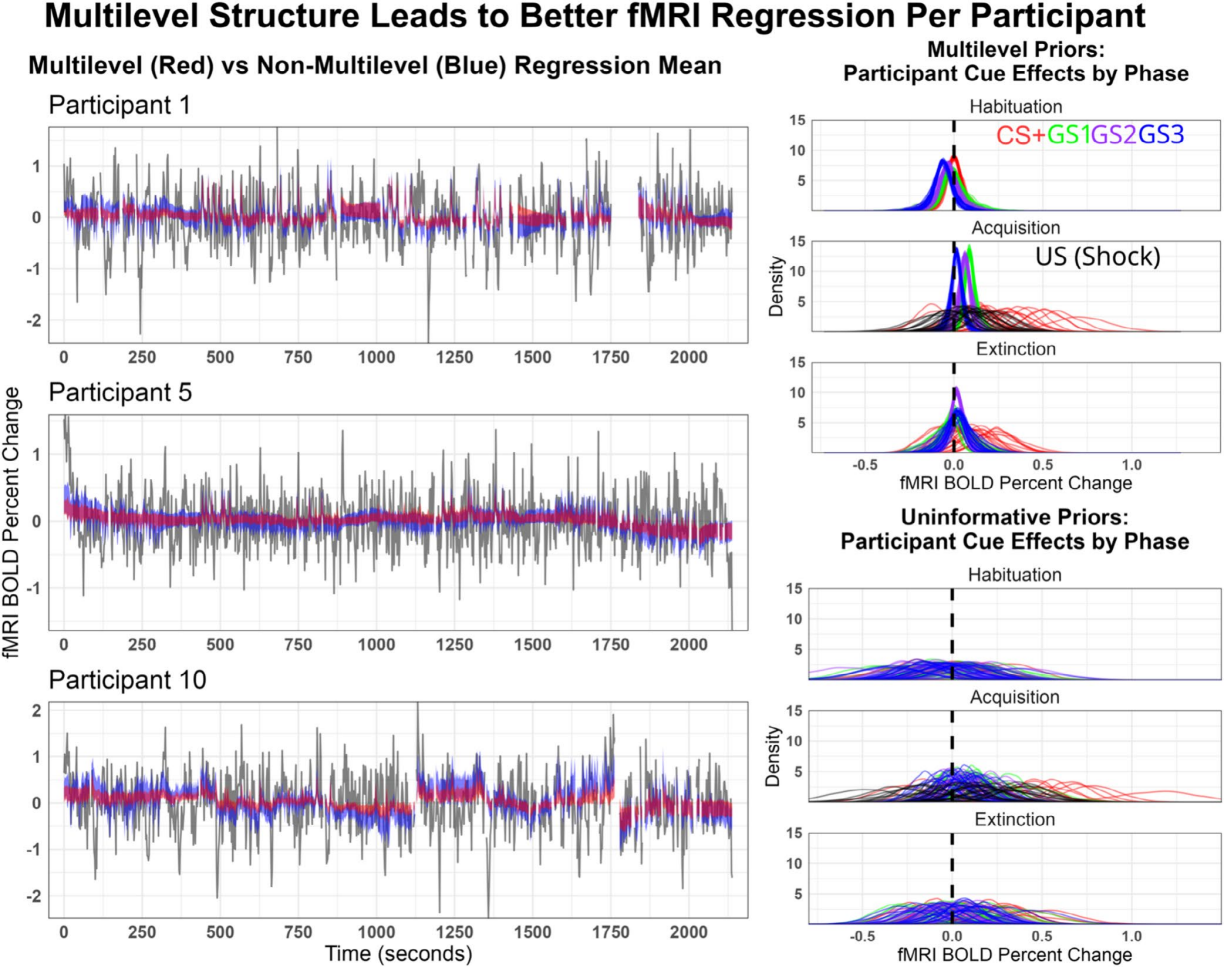
Participant‐level fMRI multiple regression of the left anterior insula with artifact contaminated data removed. The left plots show the predicted mean posterior (inner 95% density) of three participants from the two models overlaid on the observed timeseries. The red shaded ribbon used multilevel priors in which the group‐level estimates regularize participant estimates, whereas the blue ribbon did not have a multilevel structure typical of standard first‐level fMRI analyses. The right density curves depict the posterior effects of each stimulus per participant. As was expected, multilevel structure led to better cross‐validation accuracy with tighter predictions that are less susceptible to large shifts. The stimulus posterior results from the multilevel model suggest there is little variation between participants in the habituation phase, while starting in the acquisition phase, the CS+ predicts a larger anterior insula hemodynamic response that is variable between participants. The non‐multilevel results are more difficult and problematic to interpret at the participant‐level because of overfitting concerns and the validity of estimating overlapping hemodynamic responses which occurred often between the CS+ and US.

The multilevel priors improved cross‐validation accuracy for fMRI multiple regression when compared to the same model without the multilevel structure with an ELPD‐Difference of 109.3 (SE = 21.9). The multilevel model with 572 parameters had a p‐loo or effective parameters of 238.4 (SE = 3.7), whereas the non‐multilevel model estimated 528 parameters with the effective parameters being 533.4 (SE = 7.9). A visualization of the fit of the model to exemplar participants and cue effects can be found in Figure 1.

### 3.2 *Z*‐Scored ssVEP per Participant

**FIGURE 2:**
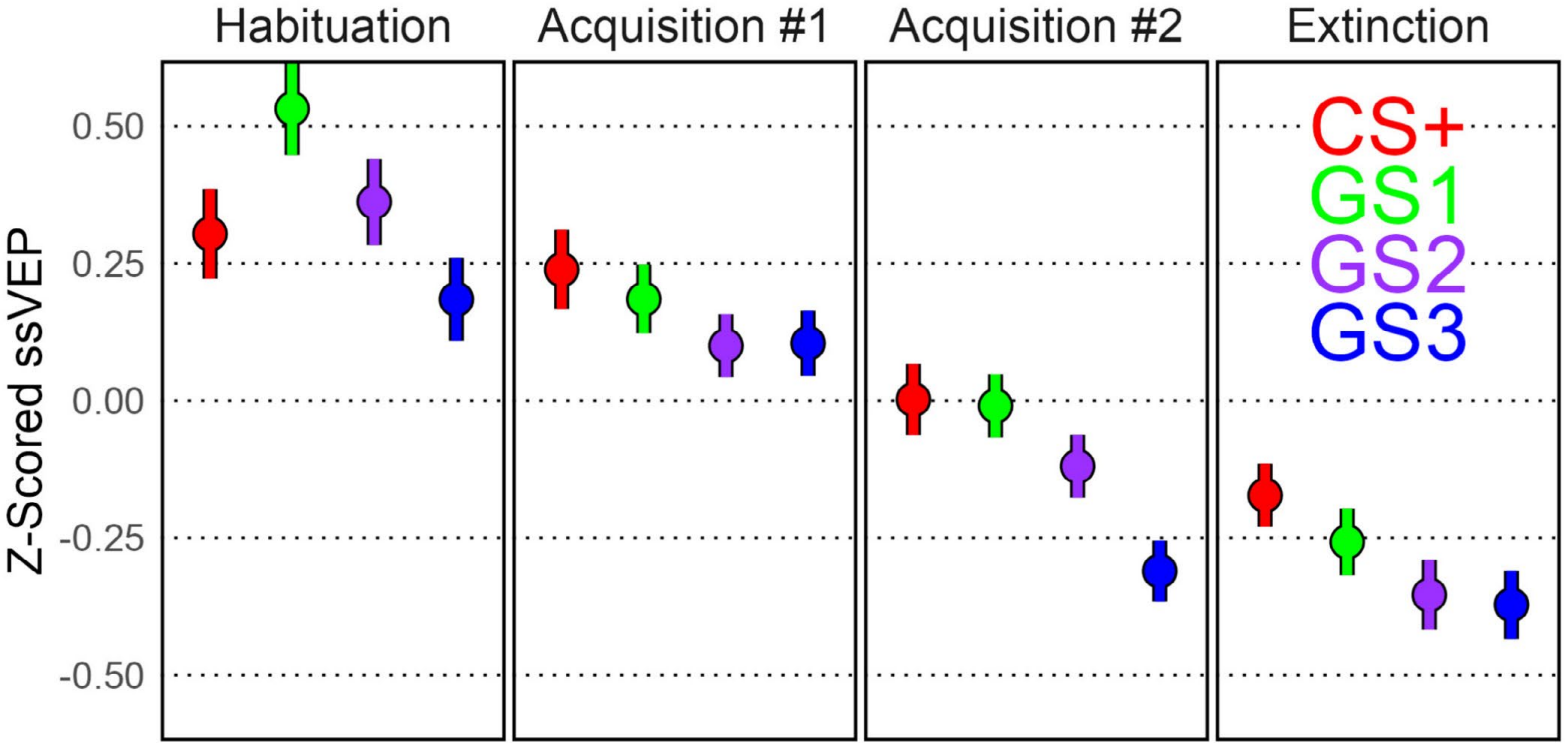
*Z*‐scored ssVEP mean and standard error per block and cue. The CS+ was paired with a noxious electric ankle shock (US) starting in the first Acquisition block at a reinforcement rate of 100% for the first 6 trials and down to 50% afterwards. The generalization stimuli (GS) were never paired with the US. The GS1 was closest in orientation to the CS+, followed by the GS2 and GS3 accordingly.

The 24 included participants had an average of 148.3 (SD = 22.6) retained trials after preprocessing. The *Z*‐scored mean ssVEP amplitude is depicted in Figure 2.

### 3.3 Model Comparison via Cross‐Validation Accuracy

The PSIS‐LOO method found the Learning Model (Model 3) had the best cross‐validation accuracy compared to Model 1 and 2, as well as all other supplemental models (Table 1). This is further shown in the model weights, in which the best cross‐validation accuracy would be attained by weighting 96% of the prediction on the Model 3. When Model 3 is excluded, the cue by block model (Model 2) outperforms the null model (Model 1) with a similar difference in model weights.

**TABLE 1:** Cross‐validation statistics comparison between models.

Models	Comparing all three models				Excluding model 3	
	Estimated parameters	Effective parameters (SE)	ELPD difference (SE)	Model weight	ELPD difference (SE)	Model weight
Model 1: Only adaptation	79	30.7 (1.2)	−63.0 (11.3)	0.00	−10.6 (4.8)	0.03
Model 2: Cue by block	88	43.0 (1.4)	−52.4 (10.6)	0.04	0.0	0.97
Model 3: Learning model	137	48.2 (1.8)	0.0	0.96		

Models	Model in Supporting Information		
	Estimated parameters	Effective parameters (SE)	ELPD difference (SE)
Model S1: 1 learning rate per participant	111	50.8 (1.9)	−17.1 (3.7)
Model S2: No invLogit of learning rates	137	49.9 (1.8)	−21.1 (4.3)
Model S3: Block arousal slope	105	34.1 (1.3)	−62.7 (10.9)
Model S4: Centered‐arousal slope	105	42.0 (1.5)	−53.4 (10.0)
Model S5: Arousal X expectancy slopes	157	50.9 (1.7)	−48.4 (10.1)

*Note:* Bayesian models were compared regarding their cross‐validation accuracy by expected log point density (ELPD), which approximates the sum of leave‐one‐out log‐likelihoods for all observations. More positive values indicate each observation is more likely and more accurately predicted if held out of model fitting. Cross‐validation accuracy can also be interpreted by model weights that indicate what linear combination of the models would be the most predictive in terms of cross‐validation accuracy. Because of the regularization of the multilevel priors, the effective number of parameters is reduced for all models. Despite the Learning Model being the most complex, it has the best cross‐validation accuracy. Both models 2 and 3 have better cross‐validation accuracy than the null model, which predicts the ssVEP with a linear adaptation over trials per participant. Five additional models were fit to the data that were not included for reasons discussed in section 2.5. For interested readers, cross‐validation relative to Model 3 is shown in the table for these supplemental models. The code and discussion of these auxiliary models is on this project's OSF page (https://osf.io/hfbjp/).

**FIGURE 3:**
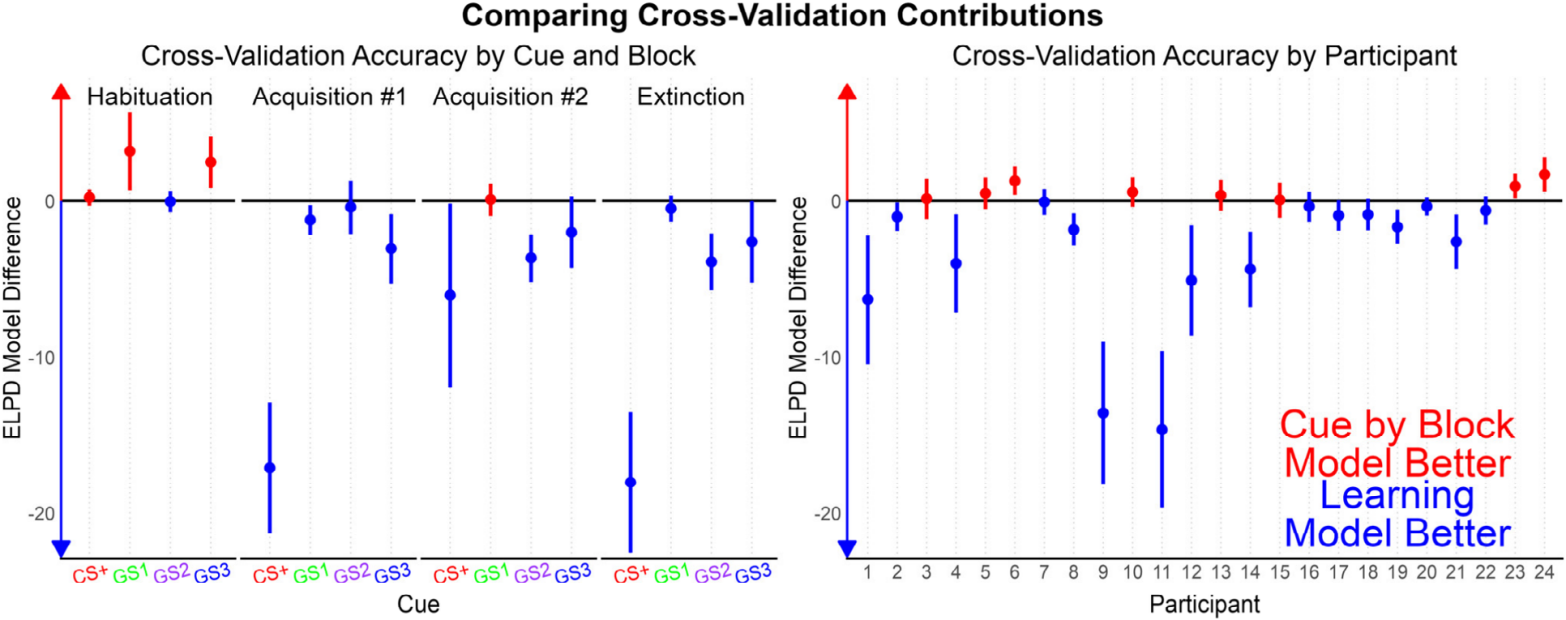
The cross‐validation measure ELPD broken down by cue, block, and by participant. ELPD is the sum of log‐likelihoods per observed data point if that data point were held out of model fitting. Visualizing the difference in ELPDs between models across relevant experimental conditions is recommended for understanding why models are more accurate and how they could possibly be improved. By subtracting the learning model (Model 3) from the cue‐by‐block model (Model 2), it becomes clear that the learning model is primarily more accurate for the CS+ cue after the habituation block and for specific participants.

A breakdown of ELPD differences by cue, block, and participant illustrates at which points Model 3 had better cross‐validation accuracy than Model 2 (Figure 3). A majority of the cues per block and participants were better predicted by Model 3. However, the bulk of Model 3's ELPD improvement was because of the CS+ predictions in blocks following habituation and for a few specific participants.

### 3.4 Posterior Analysis

The downward trend in ssVEP over trials seen in Figure 2 was confirmed via the adaptation intercept and posteriors not overlapping with zero in each of the three models: Model 1: intercept = 0.44 (SD = 0.07), slope = −0.005 (SD = 0.0007); Model 2: intercept = 0.52 (SD = 0.09), slope = −0.006 (SD = 0.001); Model 3: intercept = 0.44 (SD = 0.07), slope = −0.005 (SD = 0.0008). Because the adaptation was similar between models, it is not further described in the main text. However, a figure of the adaptation (Figure S1) and full posteriors per participant can be found in the Supporting Information.

**FIGURE 4:**
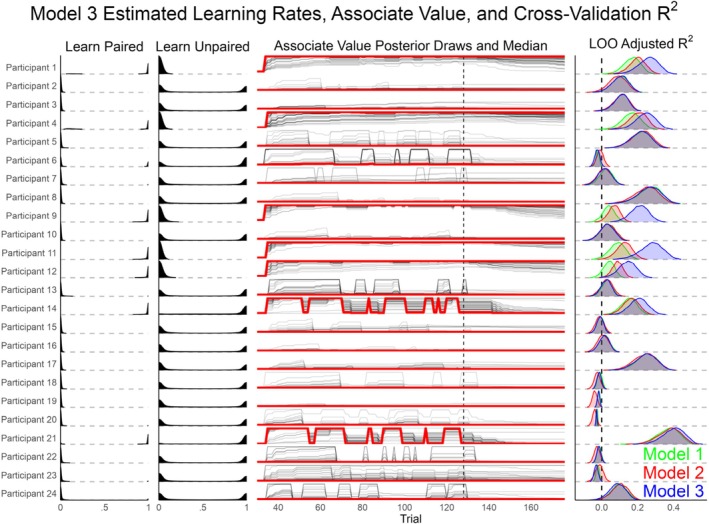
A visualization of learning rates and estimated associate value per participant. From left to right, the *LearningPaired* parameter specifies how quickly associative strength between the CS+ and US changes with each US pairing. The *LearningUnpaired* parameter indicates decreases in associate strength when the CS+ is presented without a US. The effects of these learning rates on associate strength is depicted in the third panel over trials, excluding the habituation trials where *CS + Strength* was zero. Black thin lines are 100 random posterior draws of associate strength, the red line is the median posterior out of all samples, and the vertical dashed line is the beginning of the Extinction phase. For comparison purposes, the cross‐validation LOO‐*R* ^2^ is presented for each participant between all three models. Overall, the model predicts a variety of learning patterns across participants with an increase in cross‐validation accuracy for most participants that were also predicted well by Models 1 and 2. Figure S2 in the Supporting Information presents this same information while also showing the location of paired and unpaired CS+ trials.

Model 3 posteriors relevant to associative strength learning (Figure 4; Figure S2) suggest different learning patterns between participants. The average learning rate of CS+ pairings with the US (*LearningPaired*) had a median of 0.22 (95% credibility interval: [0.01, 0.77]), whereas the *LearningUnpaired* for unpaired CS+ trials was 0.33 [0.02, 0.87]. The posterior for learning rate deviations between participants increased from a prior of Normal (−0.5, 1.5) to a mean of 2.58 (roughly normally distributed; SD = 0.87) for *σLearningPairedRaw*, and to 2.09 (SD = 0.95) for *σLearningUnpairedRaw*. This suggests that there was evidence that the learning rates between participants differed more than the chosen prior. While *CS + Strength* is not an estimated parameter, it too has a posterior dependent on draws from the two learning rates per participant. At trial 37, the first Acquisition block begins in which the first 6 CS+ trials are paired with a US (100% reinforcement rate). After these first 6 boosted trials, the CS+ is only paired with a US 50% of the time. The majority of participants have *LearningPaired* posteriors close to 0, with the *LearningUnpaired* evenly distributed at 0 and 1. These specific participants have *CS + Strength* posteriors that differ very little with each CS+ pairing. Five of the participants (1, 4, 9, 11, and 12) have *LearningPaired* rates close to 1 and *LearningUnpaired* close to 0, leading to *CS + Strength* close to 1 throughout the last three experiment blocks. Lastly, two participants (14, 21) have a *LearningPaired* posterior close to 1, but a *LearningUnpaired* posterior also close to 1. This led to large shifts in the *CS + Strength* median with each CS+ paired and unpaired trial. Also in Figure 3, the *R* ^2^ cross‐validation posteriors are visualized between the three models to contextualize each participant. A majority of participants that had little change in CS + Strength were not fit well by any of the 3 models, with LOO‐*R* ^2^ at or below zero. For participants with LOO‐*R* ^2^ above zero, Model 3 matched or exceeded the other models in cross‐validation variance explained. The overall median and 95% credibility interval LOO‐*R* ^2^ per model was: Model 1 = 0.09 [0.07, 0.11], Model 2 = 0.09 [0.07, 0.11], Model 3 = 0.12 [0.10, 0.14]. A posterior contrast suggests Model 3 explains 0.031 (0.004, 0.058) more single‐trial cross‐validation variance than Model 1 and 0.026 [−0.001, 0.053] than Model 2.

**FIGURE 5:**
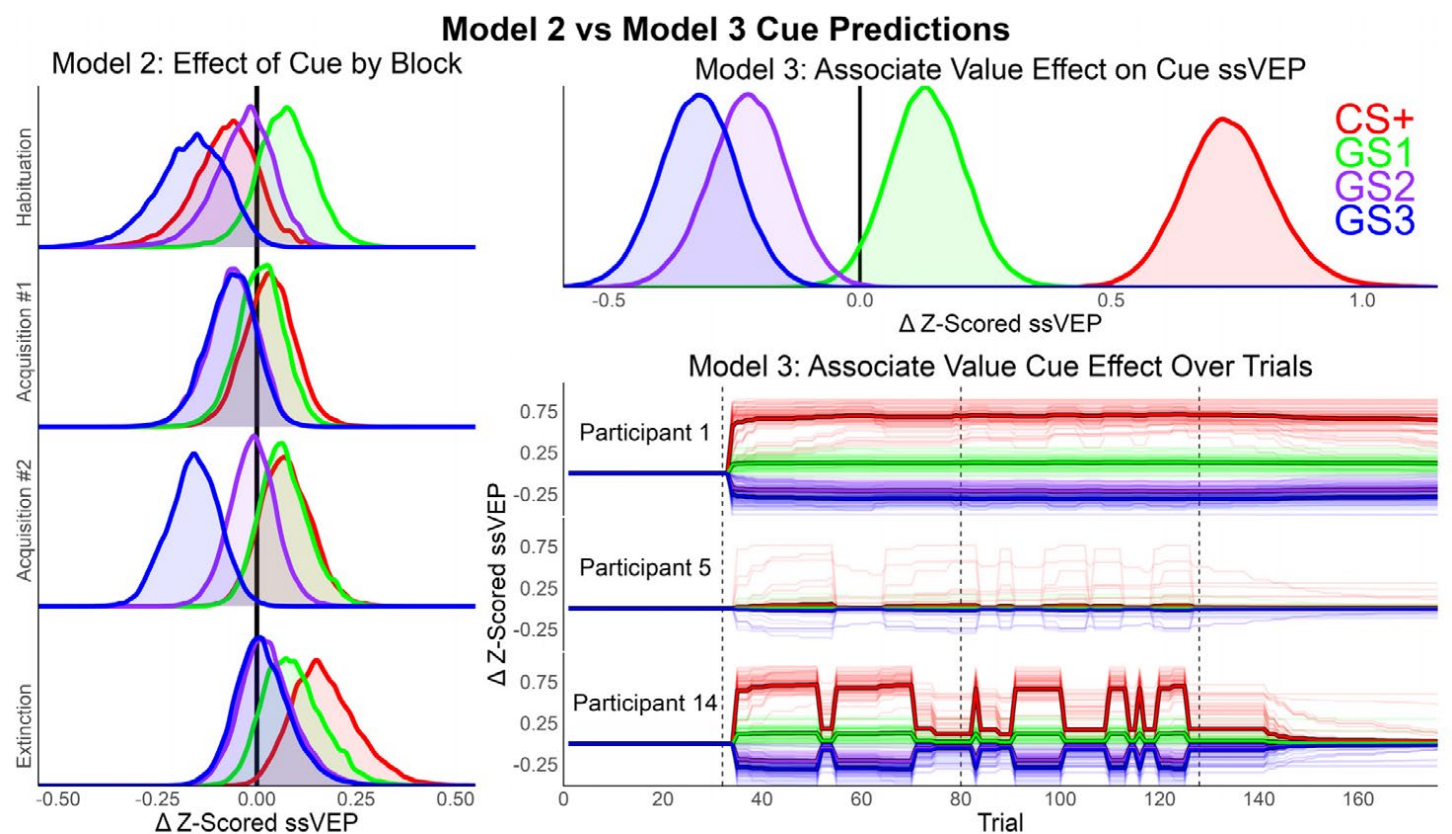
Model 2 and 3 cue relevant posteriors controlling for adaptation. Left: Posteriors for *βCue* from Model 2 represent the probability of change for the ssVEP per cue by block. Top right: The posteriors of *βScaling* from Model 3 estimate the effect on ssVEP per cue when CS+ associate strength is 1. Bottom right: The predicted effect per trial and cue (*βScaling* [1: *nCues*]⸱*CS + Strength* [*i*]) for participants 1, 5, and 14. These participants were selected because they have different patterns of learning, while still having positive LOO‐*R* ^2^ posteriors. Thin lines are 100 random posterior draws, the thicker line is the average of all samples, and vertical dashed lines indicate transitions between blocks (Habituation, Acquisition #1, Acquisition #2, and Extinction). In Model 3, the effect of cue conditioning is accentuated because it can differ over trials and between participants based on *CS + Strength* lowering statistical uncertainty.

Finally, the parameters most directly tied to differences in ssVEP cue predictions were plotted in Figure 5. *βCue* from Model 2 depicts the average uncertainty per cue per block. Alternatively, *βScaling* from Model 3 is the predicted change in cue when *CS + Strength* is at the maximum value of 1. To illustrate the complete Model 3 cue prediction, the average and 100 posterior draws for each cue prediction are depicted for three participants (Figure 5). To examine if there was evidence for aversive generalization in Model 2 and 3, a linear contrast was performed by subtracting the *βCue* and *βScaling* parameters in the expected direction from similarity to the CS+. For Model 2, the generalization posterior for Acquisition block #1 had a median of 0.14 and inner 95% density of [−0.08, 0.43]; Acquisition #2 was 0.17 [−0.07, 0.42], and Extinction was 0.04 [−0.26, 0.28]. For Model 3, the generalization posterior had a median of 1.14 [0.83, 1.50].

**FIGURE 6:**
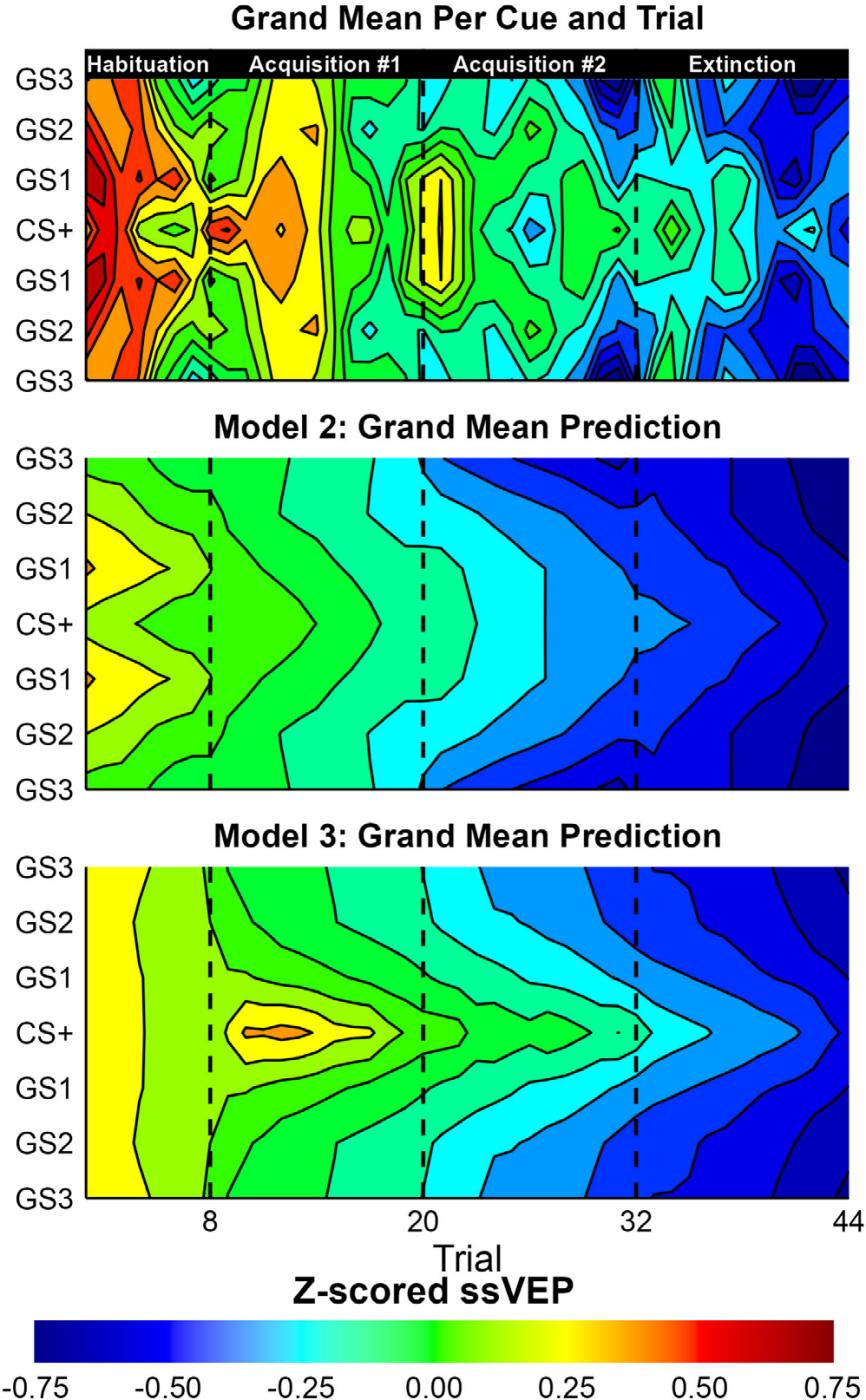
A visualization of the grand mean *Z*‐scored ssVEP observed and predicted by Model 2 and 3. The contour plot depicts the average of all trials per cue per participant. The grand means per trial were then smoothed with a moving average of two surrounding trials per cue.

Lastly, the models were used to recreate the original data. The grand mean and its model recreations are depicted in Figure 6. The correlation between observation and model predictions all had *p*‐values less than 0.001: Model 1 *r* = 0.32, *t* = 20.0; Model 2 *r* = 0.33, *t* = 21.2; Model 3 *r* = 0.37, *t* = 23.8.

## 4 Discussion

### 4.1 Summary

The present study used Bayesian multilevel models to quantify the effect of differential conditioning on fMRI‐BOLD and cue‐evoked ssVEP amplitudes as a demonstrative example of a Bayesian workflow approach. The overarching goal was to establish that a multilevel model can both establish theoretical insights while more accurately recovering aversive generalization by overcoming single‐trial noise, inter‐individual differences, and missing trials. By detailing how models are designed, compared through cross‐validation, and analyzed, this project serves as the basis for future models that will be more ambitious and informative. While the present models focus on aversive generalization conditioning, the ability to establish theoretical models with reliable and granular estimates is desirable for many fields within the neurosciences.

### 4.2 Multilevel Structure Improves fMRI First‐Level Multiple Regression

In the first models, many well‐known benefits of multilevel models are demonstrated through their application to a typical fMRI first‐level multiple regression (Figure 1). To do this, the well‐established multiple regression from AFNI's 3dREMLfit was translated to a Bayesian analog with uninformative‐weak priors. This was then compared to a second model in which multilevel priors regularize each parameter per participant. This includes not only the effect of the stimuli of interest but also the effect of movement and proportion of noise that correlated in time between volumes. This by‐default form of regularization improves the model fit per participant and cross‐validation accuracy by preventing overfitting (Gelman et al. 2012).

More importantly, the multilevel structure improved the interpretability of participant‐level posteriors in two ways. First, it becomes more apparent if there is sufficient statistical evidence of inter‐individual differences. As shown in Figure 1, there is little evidence of participant‐level cue differences in the habituation phase, which is expected given that the aversive ankle shock has not yet been paired with the CS+. After the habituation phase, there is evidence that the CS+ increases anterior insula activity, as has been previously found (Sperl et al. 2019), and that this effect varies between participants, possibly relating to important individual differences in learning rates or traits such as anxiety. The second way multilevel priors improve estimation is more specific to fMRI convolution challenges. The hemodynamic response measured by fMRI is a relatively slow process that typically peaks 9 s after excitation. This presents a problem for most conditioning studies in which the US co‐terminates with the CS+ cue, eliciting hemodynamic responses that overlap greatly. This means the response to the ankle shock could be mistakenly attributed to the cue that predicts it. For this reason, many conditioning studies only analyze unpaired trials, sacrificing statistical power. But because the multilevel structure borrows information across participants through a semi‐averaged result, such estimations of the overlapping results are made more viable and trustworthy.

### 4.3 Extending the Rescorla‐Wagner Model to EEG‐ssVEP Data

Having reiterated the benefits of multilevel priors, the EEG data was used to demonstrate how researchers can move beyond descriptive models to establish theoretical models of the underlying processes. Visible in the cue means (Figure 2), there was an overall decrease in ssVEP amplitude over trials, with greater responses to the CS+ and GS1 cues beginning in the Acquisition phase when the CS+ began being paired with the noxious US ankle shock. Three Bayesian Multilevel models were fit to the data with different predictions per trial: (1) a Null model that only accounted for the ssVEP adaptation (decrease) as a multilevel factor by participant, (2) an additional prediction for each cue per block, and (3) a Rescorla‐Wagner‐inspired learning model of associate strength that also controlled for adaptation over trials. In line with the suggested best practices for Markov Chain Bayesian models (Gelman 2014; McElreath 2020), model comparison was performed with the PSIS‐LOO cross‐validation method that approximates the log‐likelihood of each observation when held out of model fitting (Vehtari et al. 2017, 2024). While the Learning Model was the most complex (137 estimated parameters), it had the best cross‐validation accuracy, partly because of the multilevel structure constraining the degrees of freedom to lower the effective parameters to 49.2 (SE = 1.8). As is recommended for PSIS‐LOO model comparison (Vehtari et al. 2017), the ELPD per observation was segmented by experimental conditions to understand why Model 3 outperformed Model 2 in cross‐validation accuracy. While Model 3 had the best cross‐validation accuracy for the majority of experimental conditions and participants, most of its cross‐validation advantage is because of (1) predictions for the CS+ cue starting in the Acquisition phase, and (2) better fit for a subset of the participants (Figure 3).

After establishing that Model 3 is predictive and unlikely to be overfitting the data, the posteriors of interest were used to assess experimental questions. First, it was found that there was a reliable average effect of adaptation, with the ssVEP decreasing over the study (Figure S1). This average adaptation and the adaptation per participant were similar in all three models. Next, the learning rate parameters were visualized per participant (Figure 4). Although learning rates could fall anywhere between 0 and 1, the posteriors concentrated at the minimum and maximum. This resulted in three general patterns: (1) participants that had little change in *CS + Strength* (*LearningPaired* ≃ 0), (2) participants that quickly approached max *CS + Strength* with each US‐paired trial and did not decrease with unpaired trials (*LearningPaired* ≃ 1; *LearningUnpaired* ≃ 0), and (3) two participants with rapid changes in *CS + Strength* (*LearningPaired* ≃ 1; *LearningUnpaired* ≃ 1). This is shown in Figure 4 by the *CS + Strength* posterior, which is dependent on the learning rates posteriors. A relevant question for interpreting these results per participant is: are there differences in cross‐validation variance explained (LOO‐*R* ^2^) between the participants by model? If the learning Model poorly predicts observations for some participants, then the learning rates may not be interpretable. Thus, the posterior of LOO‐*R* ^2^ is plotted for each participant in Figure 4 as well. For about half of the participants, the LOO‐*R* ^2^ posteriors are at or below zero for each of the three models, and Model 3 predicts no change in *CS + Strength*. So, for these participants, where Model 3 does poorly, so do the other simpler models. For the rest of the participants that were well‐explained (LOO‐*R* ^2^ > 0), the learning model closely matches or is superior to the simpler models. There are examples of participants that are fit well for each of the three identified learning patterns, and all the participants that have a high *LearningPaired* rate are well predicted. This suggests that *CS + Strength* dependent changes in ssVEP are justifiably interpretable. Lastly, the predicted effects of conditioning on cue are visualized in Figure 5. The learning model demonstrates that there is more statistical certainty (less overlap) for how conditioning affects each cue, and it predicts a large change for each cue compared to Model 2.

It could be argued that this is a form of “cherry picking” in which we let Model 3 choose who the conditioning effect applies to, so of course it accentuates the effect of conditioning. It is true that in hindsight Model 3 applies the conditioning effect selectively to each participant, but this is not being hidden. In fact, the complete set of posterior parameters details a level of specificity and transparency ideal for scientific inference and actionable future directions. It has been argued that replicable science needs to be open and transparent, while also detailing effect sizes and even how many participants show the effect of interest (Shrout and Rodgers 2018). Here, it is clearly depicted which participants show the conditioning effect, how it changes over trials, and for which participants cross‐validation accuracy differs (ELPD) as well as a meaningful effect size measure (LOO‐*R* ^2^). Much of this information is lost in Model 2 by holding the effect of cue constant between‐participants. As shown in cross‐validation metrics, Model 2 is a less accurate depiction of each participant by over‐estimating the effect of conditioning for those that do not show the effect and underestimating it for those that do. This makes it more difficult to draw actionable insights for future directions, such as understanding why some participants do not show the effect or which learning models may better fit the underlying data. These analyses are in line with modern statistical recommendations to accurately capture the multilevel structure of the data and to represent statistical uncertainty transparently, moving away from null‐hypothesis testing (Wasserstein et al. 2019; Gelman and Brown 2024).

**FIGURE 7:**
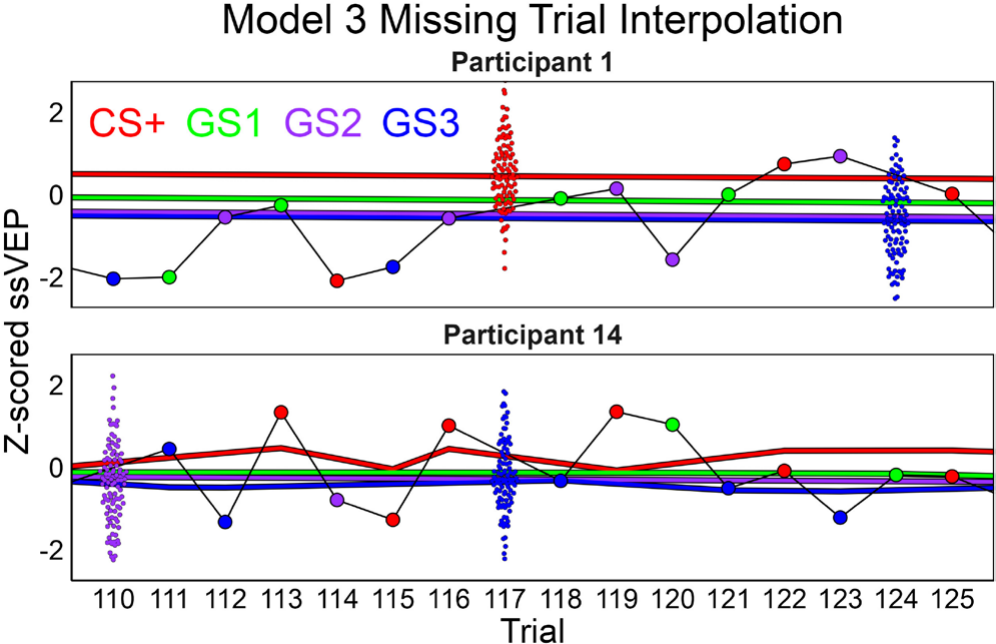
Example of missing trial interpolation for Model 3. Large dots are observed trials, thick lines are the average mean prediction per cue, and swarmed smaller dots are 100 draws for missing trials from the likelihood function. The 100 draws are plotted for clarity, but a complete analysis would use all the draws. In physiological studies, single‐trial variability is high, and it is common to lose trials for various reasons. This can have debilitating effects on statistical power, particularly for the current project that aims to correlate EEG and fMRI in dynamic learning processes. But in this Bayesian framework, missing trials are listed as parameters and are estimated by draws from the current prediction (*μ* [*i*]) and estimated single‐trial error (*σ* [participant [*i*]]). This is a justifiable replacement for a missing trial as it acts as a compromise, allowing for missing trials to still aid in model fitting while appropriately weighting them less than the actual observed trials.

Lastly, a benefit of this analysis approach is how missing trials are interpolated. It is common for trials to be lost for reasons such as of movement, eye‐blinks, or EEG sensors losing contact with the skin. When additional physiological methods are recorded simultaneously, there will be even more trials missing at least one of the measures. For difficult simultaneous EEG‐fMRI recordings, it is common to entirely lose either the EEG or fMRI recording for a participant. So while the overall objective of concurrent ssVEP‐fMRI recordings is to utilize ssVEP effects to elucidate fMRI activations, traditional methods would sacrifice statistical power and would need to exclude many participants and/or trials, hampering external validity and efficiency. But as depicted (Figure 7) in this Bayesian framework, missing trials are listed as parameters to be interpolated. The entire model is used to produce a distribution of the missing observation based on the trial's predicted mean (*μ* [*i*]) and estimated error per participant (*σ* [participant [*i*]]). In future models, in which EEG and fMRI trials are specified as coming from a multivariate/multidimensional distribution, a predicted missing trial would still inform an observed fMRI trial and vice versa, but it would be weighted appropriately less than observed trials.

The model results highlight some known qualities of the ssVEP measure, as well as some new details possibly related to the simultaneous fMRI recording. The first obvious trend in the data is a steady decrease in the overall ssVEP amplitude over trials, which we term here adaptation (Figure 2). We found that there was strong evidence for an average decrease present in nearly all participants (Figure S1; intercept and adaptation posteriors at https://osf.io/hfbjp/). It is known that the ssVEP adapts normally with repeated exposures (Norcia et al. 2015). However, the decrease in the present study is much more pronounced than has been seen in previous conditioning studies (McTeague et al. 2015; Wieser et al. 2014). The most obvious difference between this and previous studies is that participants are laying down, with simultaneous fMRI acquisition. Thus, adaptation could be accentuated by heightened sympathetic arousal at the beginning of the study (because of the loud MRI scanner) and lower arousal at the end when compared to previous EEG studies in which participants are seated in a chair. This could be represented in changes in the total global field power that may influence the recoverable ssVEP. Future research will model this spectral information, with a particular focus on alpha‐band oscillation changes, to examine how they are related to the ssVEP amplitude. However, the effect could also be partly explained by a physical interaction between the EEG system and MRI. The EEG‐system heats up during the study at the sensors and amplifier, which may affect the amplitude (Egan et al. 2021). Future research will examine if some aspect such as the gain or resistance recorded during the scan can be used to explain the adaptation effect. If these factors are present, they can be modeled explicitly in the present Bayesian framework. Lastly, it is known that a certain percentage of participants will not show an ssVEP amplitude when exposed to a flickering stimulus (Moratti et al. 2007; Regan 1989). This is likely another reason why the models do not fit well for some participants. The signal‐to‐noise ratio of the ssVEP driving frequency compared to surrounding frequencies could be used to assess the presence of an ssVEP once the other confounding factors mentioned are addressed. If a participant does not have an ssVEP, then for that participant the ssVEP should not be correlated with alpha‐oscillations, heart‐rate changes, or fMRI. Some threshold, which could be determined in a data‐driven portion of the model, should decide on a per‐participant basis if the ssVEP is informative for these other physiological measures.

### 4.4 Relevance of Contemporary Bayesian Workflows to Cognitive Neuroscience

To efficiently map many dynamic brain processes, the field needs robust single‐trial estimates from theory‐derived generative models. The benefits of using single trials for neuroimaging inferences are well‐established in the literature (Debener et al. 2006; Ullsperger 2024), but most of these analyses primarily use single trials to draw group‐level statistical inferences with a limited number of parameters. The analyses demonstrated here not only draw conclusions from single trials, but provide a justified prediction for a specific trial, cue, and participant without noise (*μ* [*i*]) or a distribution with noise (*Normal*(*μ* [*i*], *σ* [participant [*i*]])). More similar to the current approach are frequentist multilevel and structural equation models that are increasingly being used for neuroimaging data (Volpert‐Esmond et al. 2021). These models can be similarly structured and theoretically should reach similar point estimates when fit by restricted maximum likelihood. However, frequentist multilevel models can be more difficult to fit, and interpreting the parameters tends to involve the interpretation of *p*‐values that are often confused and misinterpreted (Wasserstein and Lazar 2016). Because Bayesian results are representations of the probability of a drawn sample, they are generative models capable of simulating data or interpolating missing trials. These benefits make it a compelling option, particularly when obtaining robust estimates of single trials is necessary.

More generally, cutting‐edge Bayesian innovations enable the development of explanatory models suitable for scientific inference. While Bayesian statistics and the mathematical principles of multilevel models are historically old and well‐established, only in the last 50 years have modern computing and algorithm innovations enabled their widespread use in applied science (Van De Schoot et al. 2021; Gelman and Vehtari 2021). Even still, algorithms for posterior estimation continue to improve and are packaged with expressive statistical software allowing for quick and flexible implementations of cutting‐edge statistical summaries and metrics (Štrumbelj et al. 2024). These iterative improvements in Bayesian software now allow the scaling of theory‐driven models to thousands of parameters that have complex non‐linear relationships (Gelman et al. 2015; Carpenter et al. 2017). This flexibility, paired with the logic of multilevel modeling, allows researchers to impart more of their domain expertise into their models and a pooling of information from disparate sources while controlling for confounds in detailed ways. Thus, this form of modeling is useful for causal inference in fields such as anthropology in which experiments are not possible (Deffner et al. 2022, 2024; Friederici et al. 2024). Primarily, the field of neuroimaging has utilized innovations in machine learning algorithms that maximize prediction accuracy to “decode” the complexity of the brain (Grootswagers et al. 2017). These methods are powerful exploratory approaches that can explain a large amount of cross‐validation variance and can be used for hypothesis testing when constrained in experiments. Yet a Bayesian model, in which every parameter is more deliberately chosen and interpretable, can act as a specific testable hypothesis more directly linked to a theory. In modern computer science research, the distinction between machine learning and Bayesian statistics is blurry as machine learning software such as TensorFlow can estimate posteriors (Štrumbelj et al. 2024), or alternatively, Bayesian principles are often used for deep learning algorithms and it seems likely that the most effective tools will use a combination of the approaches (Papamarkou et al. 2024). Thus, similarly, the field of neuroimaging may want to use the complementary innovations of machine learning and Bayesian statistics, as opposed to relying on interpreting decoding prediction accuracy alone, as it has been suggested to lead to misinterpretations and irreproducible findings (Hullman et al. 2022).

### 4.5 Future Directions With the Bayesian Workflow

The present project puts into practice the suggested Bayesian workflow (Gelman et al. 2020; Gabry et al. 2019; Schad et al. 2021). This is the process of iterative model building, comparison, visualization, and analysis that coincides with a formalizing and sharpening of scientific theories. Here, this involved comparing models on ELPD to gauge performance and then breaking down the models to see how they differ in their accuracy and predictions. In the present project, the interpretation was straightforward as the learning model matched the competing models or did better on all metrics. Yet, a detailed look at the model makes apparent aspects that could be improved on in the future. Future work will compare competing models of learning (e.g., Pearce‐Hall or Mackintosh models; Roesch et al. 2012) with neurophysiological informed mechanisms of attention and vision interactions (Desimone and Duncan 1995; Reynolds and Heeger 2009) to explain concurrently recorded EEG‐fMRI data. Systematic model building and comparison will be necessary to assess the complexity of these interlocking assumptions.

The present learning model was kept relatively simple for interpretability, and there are several aspects that could be improved or expanded upon in future work. The first aspect was poorer fit in the habituation phase compared to Model 2. It is possible that the orientations of different cues may naturally produce a slightly larger amplitude at different scalp locations. Thus, fitting an intercept per cue may be justified. However, it can be seen in the Supporting Information figures that there were a variety of outliers in the habituation phase, which is a likely reason Model 2 looks better during that early phase of the study because it had the flexibility to accommodate them. Separately, the adaptation slope may not be an independent or linear relationship. It may be related to the gain of the equipment used and thus could be explained quantitatively. Even so, it may not be linear and could/flatten/plateau over the course of the study. A multilevel Gaussian process regression or a spline function would allow for the modeling of adaptation to fit this phenomenon. Continuing, the scaling of CS+ associate strength per cue was done via an array per cue which did not allow for the pattern of generalization to differ between participants. Future work could combine this scaling across cues via a multilevel Ricker or Wavelet function that can fit generalization and sharpening patterns (Ahumada et al. 2025). This may improve statistical power by reducing the number of parameters to make predictions per cue. Lastly, the learning rates found suggest that the Rescorla‐Wagner may not be the best option for predicting learning for the ssVEP. The ssVEP has been tied more explicitly to attention than other physiological responses (Müller et al. 1998; Müller and Hillyard 2000; Müller, Malinowski, et al. 2003). Although learning rates could be anywhere between 0 and 1, they concentrated at the two extremes. This is why the inverse logit transformed version of the model most likely fit better than the alternative learning models, as it is a transform that is primarily used to aid in fitting binary outcomes of a logistic regression. The original Rescorla‐Wagner model would also force the use of one learning rate for paired and unpaired trials, as theoretically only the surprise related to prediction error should influence updates in learning. Thus, a different all‐or‐nothing model of learning may be better suited. Other experimental factors could have influenced this quick learning such as the long habituation phase and initial 100% pairing for the first 6 CS+ trials. This could have allowed participants to more easily differentiate the patterns and learn the relationship as other studies have found more of a gradual increase across trials (Santos‐Mayo and Moratti 2025). Nonetheless, it is informative to know such a quick learning rate can occur for the ssVEP. Other physiological measures like skin conductance or pupil diameter may take longer to be conditioned as they perhaps involve a slower process of learning.

## 5 Conclusion

This technical report sought to validate a multilevel learning model for analyzing ssVEP amplitudes during aversive conditioning, using innovations in Bayesian estimation algorithms and model comparison. Illustrating the multilevel method and its benefits was the main goal of this technical report, and we will in the future extend this work to incorporate other variables such as autonomic, behavioral, and fMRI results. We chose the present, more specific, scope for the current report because the present analysis is (1) sufficiently complex and novel to occupy a full technical report, allowing us to explain the method in detail, and (2) is based on a subset of data that is available currently. Future efforts will use the hierarchical structure to combine ssVEP and fMRI data, simultaneously collected with the data presented here.

Although the learning model examined above was relatively complex, the multilevel structure led to superior cross‐validation accuracy compared to simpler models. Going beyond previous research that used single‐trial estimates to draw meaning from a limited number of parameters, the present approach is a theory‐driven generative model providing granular and reliable estimates at the level of individual subjects, cues, and trials. Thus, it was possible to visualize individual learning rates, the associative strength between the CS+ cue and noxious US, and how this predicted ssVEP amplitudes per cue and trial. Continuing, the conditioning effects per cue were better recovered than Model 2, which held the effect of cue constant between participants. Intrinsic to this workflow is the transparency and specificity of model results, which quantify the per‐participant statistical certainty of meaningful factors such as the adaptation of the ssVEP over trials and cross‐validation variance explained. The findings provide a detailed understanding useful toward future model building efforts. Lastly, because the approach is a generative model, it was shown how missing trials can be interpolated based on the entire model's structure. This may prove essential for multimodal physiological paradigms in which simultaneously recorded measures—each with a different collection of missing trials—are fused together. Thus, Bayesian workflows as illustrated here may serve as the theoretical basis for more ambitious future work, fusing not only different imaging methods but also integrating and contrasting more complex theories of vision, attention, and aversive learning.

## Supporting information


**Figure S1:** A visualization of the adaptation posterior per model. Left: 100 posterior draws of the average adaptation. The average adaptation posteriors are used as the multilevel prior for each participant informing and regularizing the right plots. Right: for interpretability, only the median posterior draw is of each participant. All three models found strong evidence for a downward trend in the ssVEP, which was similar between participants.
**Figure S2:** Depicted are the same learning rate posteriors, CS+ associate value, and LOO‐*R* ^2^ posteriors from Model 3 in the main text. The only difference here is that in the third panel, paired (red tick marks) and unpaired (light‐blue) CS+ trials are shown along the *x*‐axis of each participant's associate value over trials. This allows for a visualization of how the CS+ trials are distributed across the acquisition and extinction phases, as well as how the model changes to paired and unpaired trials per participant.

## Data Availability

The data that support the findings of this study are openly available in Applying Bayesian multilevel modeling OSF page at https://osf.io/hfbjp/, reference number 10.17605/OSF.IO/HFBJP.

## References

[hbm70360-bib-0001] Ahrens, L. M. , P. Pauli , A. Reif , et al. 2016. “Fear Conditioning and Stimulus Generalization in Patients With Social Anxiety Disorder.” Journal of Anxiety Disorders 44: 36–46. 10.1016/j.janxdis.2016.10.003. 27728838

[hbm70360-bib-0002] Ahumada, L. , C. Panitz , C. M. Traiser , F. E. Gilbert , M. Ding , and A. Keil . 2025. “Quantifying Population‐Level Neural Tuning Functions Using Ricker Wavelets and the Bayesian Bootstrap.” Journal of Neuroscience Methods 413: 110303. 10.1016/j.jneumeth.2024.110303. 39428077 PMC12054791

[hbm70360-bib-0003] Allen, P. J. , O. Josephs , and R. Turner . 2000. “A Method for Removing Imaging Artifact From Continuous EEG Recorded During Functional MRI.” NeuroImage 12, no. 2: 230–239. 10.1006/nimg.2000.0599. 10913328

[hbm70360-bib-0004] Carpenter, B. , A. Gelman , M. D. Hoffman , et al. 2017. “Stan: A Probabilistic Programming Language.” Journal of Statistical Software 76, no. 1: 1–32. 10.18637/jss.v076.i01. 36568334 PMC9788645

[hbm70360-bib-0005] Cox, R. W. 2012. “AFNI: What a Long Strange Trip It's Been.” NeuroImage 62, no. 2: 743–747. 10.1016/j.neuroimage.2011.08.056. 21889996 PMC3246532

[hbm70360-bib-0006] Debener, S. , M. Ullsperger , M. Siegel , and A. K. Engel . 2006. “Single‐Trial EEG–fMRI Reveals the Dynamics of Cognitive Function.” Trends in Cognitive Sciences 10, no. 12: 558–563. 10.1016/j.tics.2006.09.010. 17074530

[hbm70360-bib-0007] Deffner, D. , N. Fedorova , J. Andrews , and R. McElreath . 2024. “Bridging Theory and Data: A Computational Workflow for Cultural Evolution.” Proceedings of the National Academy of Sciences of the United States of America 121, no. 48: e2322887121. 10.1073/pnas.2322887121. 39556723 PMC11621747

[hbm70360-bib-0008] Deffner, D. , J. M. Rohrer , and R. McElreath . 2022. A Causal Framework for Cross‐Cultural Generalizability. Sage.

[hbm70360-bib-0009] Desimone, R. , and J. Duncan . 1995. “Neural Mechanisms of Selective Visual Attention.” Annual Review of Neuroscience 18: 193–222. 10.1146/annurev.ne.18.030195.001205 7605061

[hbm70360-bib-0010] Duane, S. , A. D. Kennedy , B. J. Pendleton , and D. Roweth . 1987. “Hybrid Monte Carlo.” Physics Letters B 195, no. 2: 216–222. 10.1016/0370-2693(87)91197-X.

[hbm70360-bib-0011] Dunsmoor, J. E. , and G. L. Murphy . 2015. “Categories, Concepts, and Conditioning: How Humans Generalize Fear.” Trends in Cognitive Sciences 19, no. 2: 73–77. 10.1016/j.tics.2014.12.003. 25577706 PMC4318701

[hbm70360-bib-0012] Egan, M. K. , R. Larsen , J. Wirsich , B. P. Sutton , and S. Sadaghiani . 2021. “Safety and Data Quality of EEG Recorded Simultaneously With Multi‐Band fMRI.” PLoS One 16, no. 7: e0238485. 10.1371/journal.pone.0238485. 34214093 PMC8253410

[hbm70360-bib-0013] Esber, G. , G. Schoenbaum , and M. D. Iordanova . 2025. “The Rescorla‐Wagner Model: It Is Not What You Think It Is.” Neurobiology of Learning and Memory 217: 108021. 10.1016/j.nlm.2025.108021. 39805526

[hbm70360-bib-0014] Fabiani, M. , G. Gratton , and K. D. Federmeier . 2007. “Event‐Related Brain Potentials: Methods, Theory, and Applications.” In Handbook of Psychophysiology, edited by J. T. Cacioppo and L. G. Tassinary , 3rd ed., 85–119. Cambridge University Press.

[hbm70360-bib-0015] Figueira, J. S. B. , E. Kutlu , L. S. Scott , and A. Keil . 2022. “The FreqTag Toolbox: A Principled Approach to Analyzing Electrophysiological Time Series in Frequency Tagging Paradigms.” Developmental Cognitive Neuroscience 54: 101066. 10.1016/j.dcn.2022.101066. 35184025 PMC8861396

[hbm70360-bib-0016] Friederici, A. D. , R. M. Wittig , A. Anwander , et al. 2024. “Brain Structure and Function: A Multidisciplinary Pipeline to Study Hominoid Brain Evolution.” Frontiers in Integrative Neuroscience 17: 1299087. 10.3389/fnint.2023.1299087. 38260006 PMC10800984

[hbm70360-bib-0017] Gabry, J. , D. Simpson , A. Vehtari , M. Betancourt , and A. Gelman . 2019. “Visualization in Bayesian Workflow.” Journal of the Royal Statistical Society: Series A (Statistics in Society) 182, no. 2: 389–402. 10.1111/rssa.12378.

[hbm70360-bib-0018] Gelman, A. 2014. Bayesian Data Analysis. 3rd ed. CRC Press.

[hbm70360-bib-0086] Gelman, A. , and N. J. Brown . 2024. “How Statistical Challenges and Misreadings of the Literature Combine to Produce Unreplicable Science: An Example From Psychology.” Advances in Methods and Practices in Psychological Science 7, no. 4: 25152459241276398. 10.1177/25152459241276398.

[hbm70360-bib-0020] Gelman, A. , J. B. Carlin , H. S. Stern , D. B. Dunson , A. Vehtari , and D. B. Rubin . 2014. Bayesian Data Analysis Third Edition (With Errors Fixed as of 15 February 2021). Chapman and Hall/CRC.

[hbm70360-bib-0022] Gelman, A. , J. Hill , and M. Yajima . 2012. “Why We (Usually) Don't Have to Worry About Multiple Comparisons.” Journal of Research on Educational Effectiveness 5, no. 2: 189–211. 10.1080/19345747.2011.618213.

[hbm70360-bib-0023] Gelman, A. , D. Lee , and J. Guo . 2015. “Stan: A Probabilistic Programming Language for Bayesian Inference and Optimization.” Journal of Educational and Behavioral Statistics 40, no. 5: 530–543. 10.3102/1076998615606113.

[hbm70360-bib-0024] Gelman, A. , and A. Vehtari . 2021. “What Are the Most Important Statistical Ideas of the Past 50 Years?” Journal of the American Statistical Association 116, no. 536: 2087–2097. 10.1080/01621459.2021.1938081.

[hbm70360-bib-0025] Gelman, A. , A. Vehtari , D. Simpson , et al. 2020. “Bayesian Workflow (No. arXiv:2011.01808).” arXiv. 10.48550/arXiv.2011.01808.

[hbm70360-bib-0026] Glasser, M. F. , T. S. Coalson , E. C. Robinson , et al. 2016. “A Multi‐Modal Parcellation of Human Cerebral Cortex.” Nature 536, no. 7615: 171–178. 10.1038/nature18933. 27437579 PMC4990127

[hbm70360-bib-0027] Grootswagers, T. , S. G. Wardle , and T. A. Carlson . 2017. “Decoding Dynamic Brain Patterns From Evoked Responses: A Tutorial on Multivariate Pattern Analysis Applied to Time Series Neuroimaging Data.” Journal of Cognitive Neuroscience 29, no. 4: 677–697. 10.1162/jocn_a_01068. 27779910

[hbm70360-bib-0028] Hedge, C. , G. Powell , and P. Sumner . 2018. “The Reliability Paradox: Why Robust Cognitive Tasks Do Not Produce Reliable Individual Differences.” Behavior Research Methods 50, no. 3: 1166–1186. 10.3758/s13428-017-0935-1. 28726177 PMC5990556

[hbm70360-bib-0029] Hoffman, M. D. , and A. Gelman . 2014. “The No‐U‐Turn Sampler: Adaptively Setting Path Lengths in Hamiltonian Monte Carlo.” Journal of Machine Learning Research 15: 1593–1623.

[hbm70360-bib-0030] Hullman, J. , S. Kapoor , P. Nanayakkara , A. Gelman , and A. Narayanan . 2022. “The Worst of Both Worlds: A Comparative Analysis of Errors in Learning From Data in Psychology and Machine Learning.” Proceedings of the 2022 AAAI/ACM Conference on AI, Ethics, and Society: 335–348. 10.1145/3514094.3534196.

[hbm70360-bib-0032] Kuhn, T. S. 1997. The Structure of Scientific Revolutions. Vol. 962. University of Chicago Press.

[hbm70360-bib-0084] Lakatos, I. 1970. “History of Science and Its Rational Reconstructions.” In PSA: Proceedings of the Biennial Meeting of the Philosophy of Science Association, vol. 1970, 91–136. Cambridge University Press. 10.1086/psaprocbienmeetp.1970.495757.

[hbm70360-bib-0034] Li, W. , and A. Keil . 2023. “Sensing Fear: Fast and Precise Threat Evaluation in Human Sensory Cortex.” Trends in Cognitive Sciences 27, no. 4: 341–352. 10.1016/j.tics.2023.01.001. 36732175 PMC10023404

[hbm70360-bib-0035] Lissek, S. 2012. “Toward an Account of Clinical Anxiety Predicated on Basic, Neurally Mapped Mechanisms of Pavlovian Fear‐Learning: The Case for Conditioned Overgeneralization.” Depression and Anxiety 29, no. 4: 4. 10.1002/da.21922. 22447565 PMC4194209

[hbm70360-bib-0036] Lissek, S. , D. E. Bradford , R. P. Alvarez , et al. 2014. “Neural Substrates of Classically Conditioned Fear‐Generalization in Humans: A Parametric fMRI Study.” Social Cognitive and Affective Neuroscience 9, no. 8: 8. 10.1093/scan/nst096. PMC4127021 23748500

[hbm70360-bib-0037] Lissek, S. , J. Levenson , A. L. Biggs , et al. 2008. “Elevated Fear Conditioning to Socially Relevant Unconditioned Stimuli in Social Anxiety Disorder.” American Journal of Psychiatry 165, no. 1: 124–132. 10.1176/appi.ajp.2007.06091513. 18006874 PMC2538574

[hbm70360-bib-0040] Liu, Y. , A. Keil , and M. Ding . 2012. “Effects of Emotional Conditioning on Early Visual Processing: Temporal Dynamics Revealed by ERP Single‐Trial Analysis.” Human Brain Mapping 33, no. 4: 4. 10.1002/hbm.21259. PMC6870142 21500315

[hbm70360-bib-0041] Lonsdorf, T. B. , M. M. Menz , M. Andreatta , et al. 2017. “Don't Fear ‘Fear Conditioning’: Methodological Considerations for the Design and Analysis of Studies on Human Fear Acquisition, Extinction, and Return of Fear.” Neuroscience and Biobehavioral Reviews 77: 247–285. 10.1016/j.neubiorev.2017.02.026. 28263758

[hbm70360-bib-0042] Lopez‐Calderon, J. , and S. J. Luck . 2014. “ERPLAB: An Open‐Source Toolbox for the Analysis of Event‐Related Potentials.” Frontiers in Human Neuroscience 8: 213. 10.3389/fnhum.2014.00213. 24782741 PMC3995046

[hbm70360-bib-0043] McElreath, R. 2020. Statistical Rethinking: A Bayesian Course With Examples in R and Stan. 2nd ed. Chapman and Hall/CRC. 10.1201/9780429029608.

[hbm70360-bib-0044] McTeague, L. M. , L. F. Gruss , and A. Keil . 2015. “Aversive Learning Shapes Neuronal Orientation Tuning in Human Visual Cortex.” Nature Communications 6: 7823. 10.1038/ncomms8823. PMC4518478 26215466

[hbm70360-bib-0045] Moratti, S. , B. A. Clementz , Y. Gao , T. Ortiz , and A. Keil . 2007. “Neural Mechanisms of Evoked Oscillations: Stability and Interaction With Transient Events.” Human Brain Mapping 28, no. 12: 1318–1333. 10.1002/hbm.20342. 17274017 PMC6871406

[hbm70360-bib-0046] Morgan, S. T. , J. C. Hansen , and S. A. Hillyard . 1996. “Selective Attention to Stimulus Location Modulates the Steady‐State Visual Evoked Potential.” Proceedings of the National Academy of Sciences of the United States of America 93, no. 10: 10. 10.1073/pnas.93.10.4770 PMC39354 8643478

[hbm70360-bib-0047] Müller, M. M. , and S. Hillyard . 2000. “Concurrent Recording of Steady‐State and Transient Event‐Related Potentials as Indices of Visual‐Spatial Selective Attention.” Clinical Neurophysiology 111, no. 9: 1544–1552. 10.1016/S1388-2457(00)00371-0. 10964063

[hbm70360-bib-0048] Müller, M. M. , P. Malinowski , T. Gruber , and S. A. Hillyard . 2003. “Sustained Division of the Attentional Spotlight.” Nature 424: 6946. http://www.ncbi.nlm.nih.gov/entrez/query.fcgi?cmd=Retrieve&db=PubMed&dopt=Citation&list_uids=12867981. 10.1038/nature01812 12867981

[hbm70360-bib-0049] Müller, M. M. , T. W. Picton , P. Valdes‐Sosa , J. Riera , W. A. Teder‐Salejarvi , and S. A. Hillyard . 1998. “Effects of Spatial Selective Attention on the Steady‐State Visual Evoked Potential in the 20–28 Hz Range.” Brain Research. Cognitive Brain Research 6, no. 4: 4. http://www.ncbi.nlm.nih.gov/cgi‐bin/Entrez/referer?http://www.elsevier.com:80/cgi‐bin/cas/tree/store/bresc/cas_sub/browse/browse.cgi%3fyear=1998&volume=6&issue=4&aid=30128. 10.1016/s0926-6410(97)00036-0 9593922

[hbm70360-bib-0050] Niazy, R. K. , C. F. Beckmann , G. D. Iannetti , J. M. Brady , and S. M. Smith . 2005. “Removal of FMRI Environment Artifacts From EEG Data Using Optimal Basis Sets.” NeuroImage 28, no. 3: 720–737. 10.1016/j.neuroimage.2005.06.067. 16150610

[hbm70360-bib-0051] Norcia, A. M. , L. G. Appelbaum , J. M. Ales , B. R. Cottereau , and B. Rossion . 2015. “The Steady‐State Visual Evoked Potential in Vision Research: A Review.” Journal of Vision 15, no. 6: 4. 10.1167/15.6.4. PMC4581566 26024451

[hbm70360-bib-0052] Papamarkou, T. , M. Skoularidou , K. Palla , et al. 2024. “Position: Bayesian Deep Learning is Needed in the Age of Large‐Scale AI.”

[hbm70360-bib-0054] Petro, N. M. , L. F. Gruss , S. Yin , et al. 2017. “Multimodal Imaging Evidence for a Frontoparietal Modulation of Visual Cortex During the Selective Processing of Conditioned Threat.” Journal of Cognitive Neuroscience 29, no. 6: 953–967. 10.1162/jocn_a_01114. 28253082 PMC5529037

[hbm70360-bib-0080] Petro, N. M. , and A. Keil . 2015. “Pre‐Target Oscillatory Brain Activity and the Attentional Blink.” Experimental Brain Research 233, no. 12: 3583–3595. 10.1007/s00221-015-4418-2. 26341931 PMC4651748

[hbm70360-bib-0055] Popper, K. 2005. The Logic of Scientific Discovery. Routledge.

[hbm70360-bib-0081] Regan, D. 1972. Evoked Potentials in Psychology, Sensory Physiology and Clinical Medicine, 328. Chapman and Hall.

[hbm70360-bib-0056] Regan, D. 1989. Human Brain Electrophysiology: Evoked Potentials and Evoked Magnetic Fields in Science and Medicine. Elsevier.

[hbm70360-bib-0082] Rescorla, R. A. , and A. R. Wagner . 1972. “A Theory of Pavlovian Conditioning: Variations in the Effectiveness of Reinforcement and Nonreinforcement.” In Classical Conditioning II, edited by A. H. Black and W. F. Prokasy , 64–99. Appleton‐Century‐Crofts.

[hbm70360-bib-0057] Reynolds, J. H. , and D. J. Heeger . 2009. “The Normalization Model of Attention.” Neuron 61, no. 2: 168–185. 10.1016/j.neuron.2009.01.002. 19186161 PMC2752446

[hbm70360-bib-0058] Reynolds, R. C. , D. R. Glen , G. Chen , Z. S. Saad , R. W. Cox , and P. A. Taylor . 2024. “Processing, Evaluating, and Understanding FMRI Data With afni_proc. Py.” Imaging Neuroscience 2: 1–52. 10.1162/imag_a_00347. PMC11576932 39575179

[hbm70360-bib-0059] Reynolds, R. C. , P. A. Taylor , and D. R. Glen . 2023. “Quality Control Practices in FMRI Analysis: Philosophy, Methods and Examples Using AFNI.” Frontiers in Neuroscience 16: 1073800. 10.3389/fnins.2022.1073800. 36793774 PMC9922690

[hbm70360-bib-0060] Roesch, M. R. , G. R. Esber , J. Li , N. D. Daw , and G. Schoenbaum . 2012. “Surprise! Neural Correlates of Pearce–Hall and Rescorla–Wagner Coexist Within the Brain.” European Journal of Neuroscience 35, no. 7: 1190–1200. 10.1111/j.1460-9568.2011.07986.x. 22487047 PMC3325511

[hbm70360-bib-0061] Santos‐Mayo, A. , and S. Moratti . 2025. “How Fear Conditioning Affects the Visuocortical Processing of Context Cues in Humans. Evidence From Steady State Visual Evoked Responses.” Cortex 183: 21–37. 10.1016/j.cortex.2024.11.005. 39608048

[hbm70360-bib-0062] Schad, D. J. , M. Betancourt , and S. Vasishth . 2021. “Toward a Principled Bayesian Workflow in Cognitive Science.” Psychological Methods 26, no. 1: 103–126. 10.1037/met0000275. 32551748

[hbm70360-bib-0063] Shrout, P. E. , and J. L. Rodgers . 2018. “Psychology, Science, and Knowledge Construction: Broadening Perspectives From the Replication Crisis.” Annual Review of Psychology 69, no. 1: 487–510. 10.1146/annurev-psych-122216-011845. 29300688

[hbm70360-bib-0064] Shuler, M. G. , and M. F. Bear . 2006. “Reward Timing in the Primary Visual Cortex.” Science 311: 5767. http://www.ncbi.nlm.nih.gov/entrez/query.fcgi?cmd=Retrieve&db=PubMed&dopt=Citation&list_uids=16543459. 10.1126/science.1123513 16543459

[hbm70360-bib-0085] Soto, F. A. , E. H. Vogel , Y. E. Uribe‐Bahamonde , and O. D. Perez . 2023. “Why Is the Rescorla‐Wagner Model So Influential?” Neurobiology of Learning and Memory 204: 107794. 10.1016/j.nlm.2023.107794. 37473985

[hbm70360-bib-0083] Sperl, M. F. , C. Panitz , I. M. Rosso , et al. 2019. “Fear Extinction Recall Modulates Human Frontomedial Theta and Amygdala Activity.” Cerebral Cortex 29, no. 2: 701–715. 10.1093/cercor/bhx353. 29373635 PMC6659170

[hbm70360-bib-0065] Stan Development Team . 2024. “Stan Modeling Language Users Guide and Reference Manual (Version 2.4.3).” [Computer Software]. https://mc‐stan.org.

[hbm70360-bib-0066] Štrumbelj, E. , A. Bouchard‐Côté , J. Corander , et al. 2024. “Past, Present and Future of Software for Bayesian Inference.” Statistical Science 39, no. 1: 376–379. 10.1214/23-STS907.

[hbm70360-bib-0067] Tran, Y. , R. A. Thuraisingham , A. Craig , and H. Nguyen . 2009. “Evaluating the Efficacy of an Automated Procedure for EEG Artifact Removal.” Conference Proceedings: Annual International Conference of the IEEE Engineering in Medicine and Biology Society 2009: 376–379. 10.1109/IEMBS.2009.5334554. 19964930

[hbm70360-bib-0068] Ullsperger, M. 2024. “Beyond Peaks and Troughs: Multiplexed Performance Monitoring Signals in the EEG.” Psychophysiology 61, no. 7: e14553. 10.1111/psyp.14553. 38415791

[hbm70360-bib-0069] Van De Schoot, R. , S. Depaoli , R. King , et al. 2021. “Bayesian Statistics and Modelling.” Nature Reviews Methods Primers 1, no. 1: 1. 10.1038/s43586-020-00001-2.

[hbm70360-bib-0070] Vehtari, A. , A. Gelman , and J. Gabry . 2017. “Practical Bayesian Model Evaluation Using Leave‐One‐Out Cross‐Validation and WAIC.” Statistics and Computing 27, no. 5: 1413–1432. 10.1007/s11222-016-9696-4.

[hbm70360-bib-0071] Vehtari, A. , D. Simpson , A. Gelman , Y. Yao , and J. Gabry . 2024. “Pareto Smoothed Importance Sampling.” Journal of Machine Learning Research 25: 1–58.

[hbm70360-bib-0072] Volpert‐Esmond, H. I. , E. Page‐Gould , and B. D. Bartholow . 2021. “Using Multilevel Models for the Analysis of Event‐Related Potentials.” International Journal of Psychophysiology 162: 145–156. 10.1016/j.ijpsycho.2021.02.006. 33600841 PMC8050933

[hbm70360-bib-0073] Wasserstein, R. L. , and N. A. Lazar . 2016. “The ASA Statement on *p* ‐Values: Context, Process, and Purpose.” American Statistician 70, no. 2: 129–133. 10.1080/00031305.2016.1154108.

[hbm70360-bib-0074] Wasserstein, R. L. , A. L. Schirm , and N. A. Lazar . 2019. “Moving to a World Beyond “*p* < 0.05”.” American Statistician 73, no. sup1: 1–19. 10.1080/00031305.2019.1583913.

[hbm70360-bib-0075] Wieser, M. J. , and A. Keil . 2011. “Temporal Trade‐Off Effects in Sustained Attention: Dynamics in Visual Cortex Predict the Target Detection Performance During Distraction.” Journal of Neuroscience 31, no. 21: 7784–7790. 10.1523/JNEUROSCI.5632-10.2011. 21613491 PMC3123450

[hbm70360-bib-0076] Wieser, M. J. , and A. Keil . 2020. “Attentional Threat Biases and Their Role in Anxiety: A Neurophysiological Perspective.” International Journal of Psychophysiology 153: 148–158. 10.1016/j.ijpsycho.2020.05.004. 32428525

[hbm70360-bib-0077] Wieser, M. J. , V. Miskovic , and A. Keil . 2016. “Steady‐State Visual Evoked Potentials as a Research Tool in Social Affective Neuroscience.” Psychophysiology 53, no. 12: 1763–1775. 10.1111/psyp.12768. 27699794 PMC5582350

[hbm70360-bib-0078] Wieser, M. J. , V. Miskovic , S. Rausch , and A. Keil . 2014. “Different Time Course of Visuocortical Signal Changes to Fear‐Conditioned Faces With Direct or Averted Gaze: A ssVEP Study With Single‐Trial Analysis.” Neuropsychologia 62: 101–110. 10.1016/j.neuropsychologia.2014.07.009. 25050854

[hbm70360-bib-0079] Wieser, M. J. , P. Reicherts , G. Juravle , and A. von Leupoldt . 2016. “Attention Mechanisms During Predictable and Unpredictable Threat—A Steady‐State Visual Evoked Potential Approach.” NeuroImage 139: 167–175. 10.1016/j.neuroimage.2016.06.026. 27318217

