# Supplementary material for: Applying Bayesian Multilevel Modeling to Single Trial Dynamics: A Demonstration in Aversive Conditioning
Source: Hum Brain Mapp. 2025 Sep 27;46(14):e70360. doi: 10.1002/hbm.70360 (PMC12476001; doi:10.1002/hbm.70360)
Supplement: Supplementary file 1 — Figure S1: A visualization of the adaptation posterior per model. Left: 100 posterior draws of the average adaptation. The average adaptation posteriors are used as the multilevel prior for each participant informing and regularizing the right plots. Right: for interpretability, only the median posterior draw is of each participant. All three models found strong evidence for a downward trend in the ssVEP, which was similar between participants. Figure S2: Depicted are the same learning rate posteriors, CS+ associate value, and LOO‐R 2 posteriors from Model 3 in the main text. The only difference here is that in the third panel, paired (red tick marks) and unpaired (light‐blue) CS+ trials are shown along the x‐axis of each participant's associate value over trials. This allows for a visualization of how the CS+ trials are distributed across the acquisition and extinction phases, as well as how the model changes to paired and unpaired trials per participant. [file HBM-46-e70360-s001.docx]

**Supplemental materials**

The code for preprocessing, models, analyses, figures can be found on the OSF page (<https://osf.io/hfbjp/>). The Farkas_Gaborgen_EEG_method_paper_equations.html details the rationale for prior selection and information on the 5 auxiliary models fit but not presented in the manuscript. Moving this information to a supplemental document was done to enhance transparency while keeping the manuscript readable. The statistical results for the adaptation decrease is presented below in Figure S1. There, 100 posterior draws are shown for the average adaptation as well as the median per participant per model. The median is shown per participant to aid in visualizing the difference between-participants. The full posterior adaptation intercept and slope per model can be found on the OSF page.


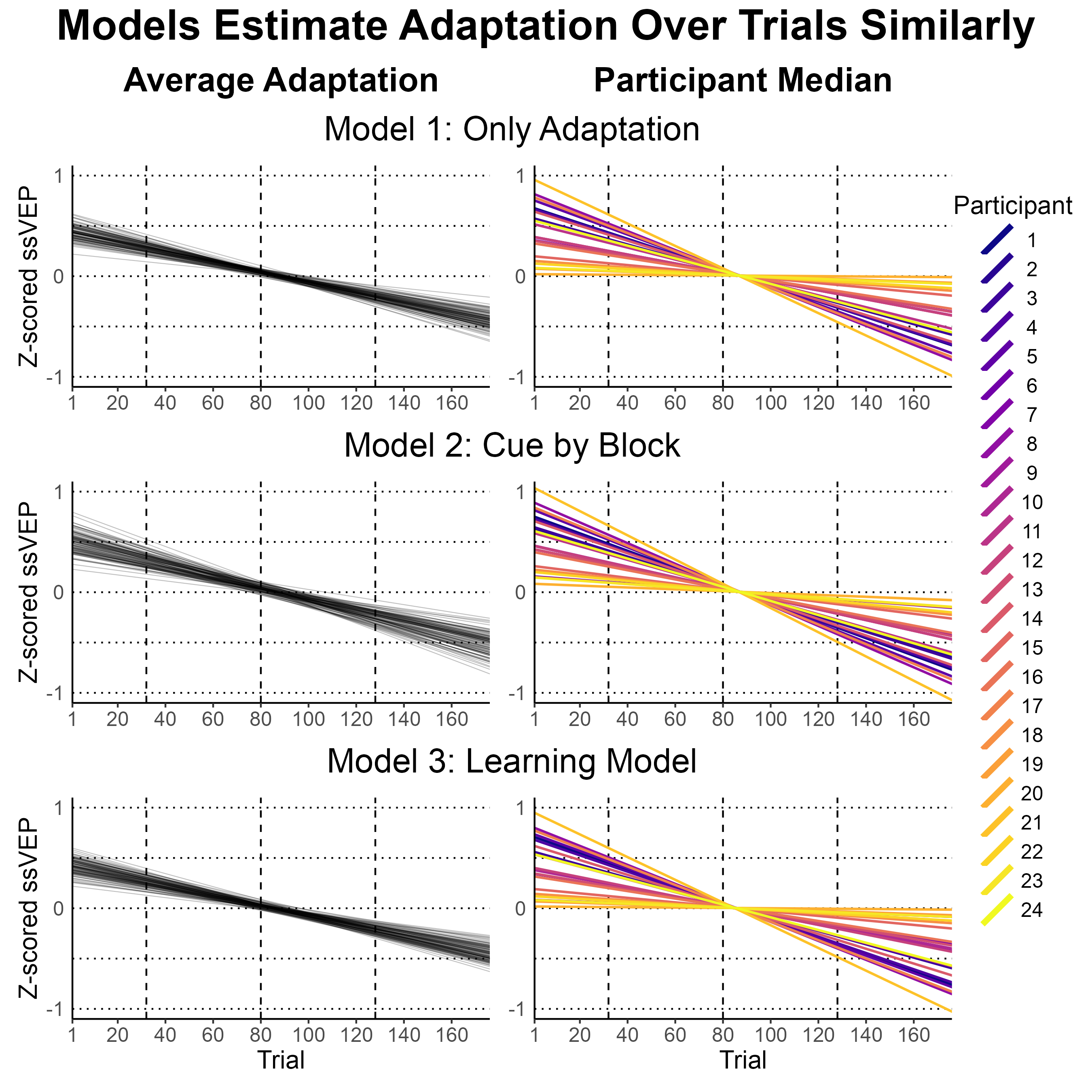


**Supplemental Figure S1.**  A visualization of the adaptation posterior per model. Left: 100 posterior draws of the average adaptation. The average adaptation posteriors are used as the multilevel prior for each participant informing and regularizing the right plots. Right: for interpretability, only the median posterior draw is of each participant. All three models found strong evidence for a downward trend in the ssVEP, which was similar between participants.


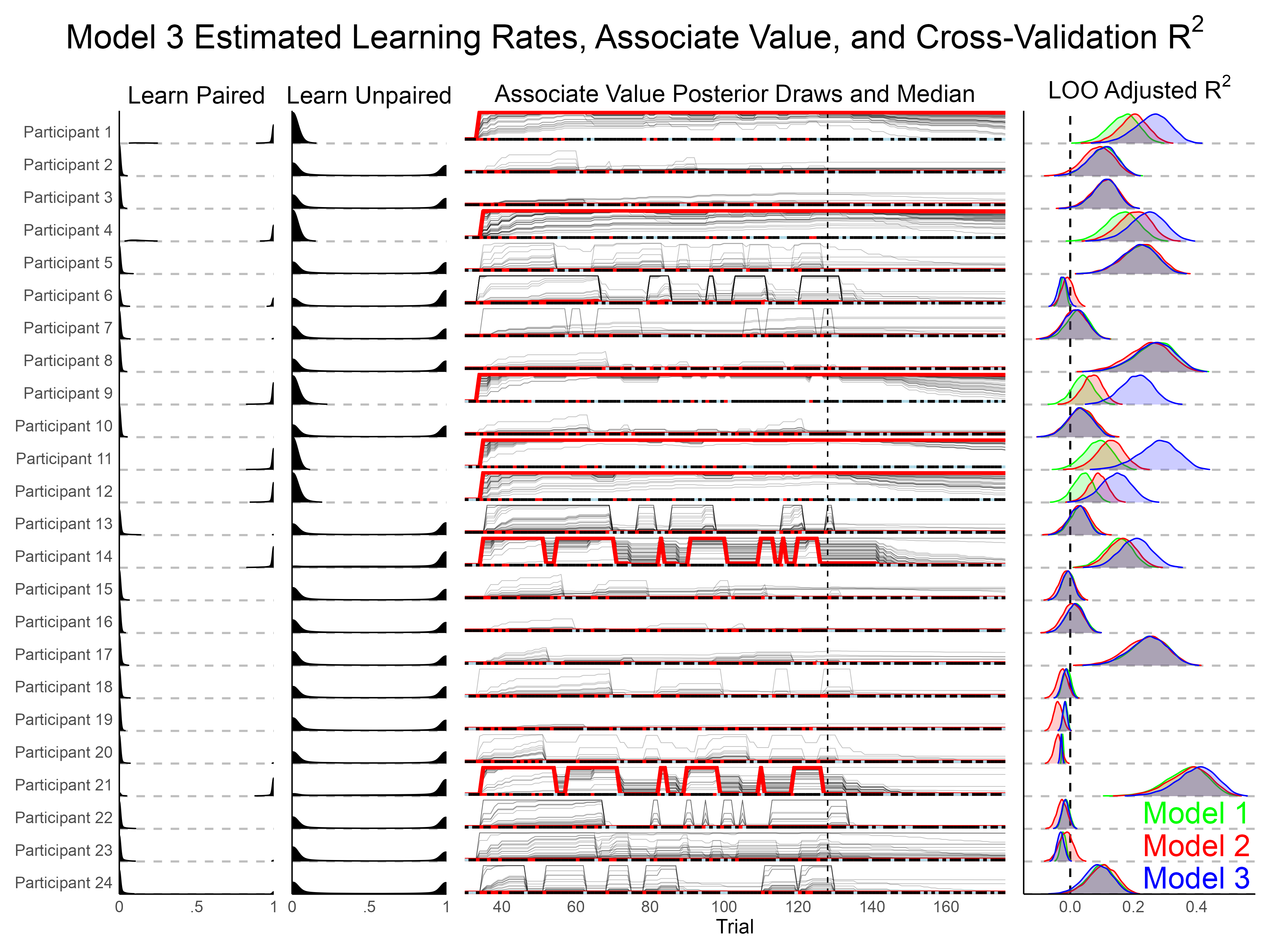


**Supplemental Figure S2.** Depicted are the same learning rate posteriors, CS+ associate value, and LOO-*R^2^* posteriors from Model 3 in the main text. The only difference here is that in the third panel, paired (red tick marks) and unpaired (light-blue) CS+ trials are shown along the x-axis of each participant’s associate value over trials. This allows for a visualization of how the CS+ trials are distributed across the acquisition and extinction phases, as well as how the model changes to paired and unpaired trials per participant.
